# metaXplor: an interactive viral and microbial metagenomic data manager

**DOI:** 10.1093/gigascience/giab001

**Published:** 2021-02-02

**Authors:** Guilhem Sempéré, Adrien Pétel, Magsen Abbé, Pierre Lefeuvre, Philippe Roumagnac, Frédéric Mahé, Gaël Baurens, Denis Filloux

**Affiliations:** CIRAD, UMR INTERTRYP, F-34398 Montpellier, France; South Green Bioinformatics Platform, Bioversity, CIRAD, INRAE, IRD, Montpellier, France; INTERTRYP, Université de Montpellier, CIRAD, IRD, 34398 Montpellier, France; CIRAD, UMR PVBMT, F-97410 St Pierre, La Réunion, France; CIRAD, UMR INTERTRYP, F-34398 Montpellier, France; INTERTRYP, Université de Montpellier, CIRAD, IRD, 34398 Montpellier, France; CIRAD, UMR PVBMT, F-97410 St Pierre, La Réunion, France; CIRAD, BGPI, 34398 Montpellier, France; BGPI, INRAE, CIRAD, Institut Agro, Université de Montpellier, 34398 Montpellier, France; CIRAD, BGPI, 34398 Montpellier, France; BGPI, INRAE, CIRAD, Institut Agro, Université de Montpellier, 34398 Montpellier, France; CIRAD, UMR INTERTRYP, F-34398 Montpellier, France; INTERTRYP, Université de Montpellier, CIRAD, IRD, 34398 Montpellier, France; CIRAD, BGPI, 34398 Montpellier, France; BGPI, INRAE, CIRAD, Institut Agro, Université de Montpellier, 34398 Montpellier, France

**Keywords:** metagenomics, metabarcoding, shotgun, sample, sequence, assignment, taxonomy, web, NoSQL, data management

## Abstract

**Background:**

Efficiently managing large, heterogeneous data in a structured yet flexible way is a challenge to research laboratories working with genomic data. Specifically regarding both shotgun- and metabarcoding-based metagenomics, while online reference databases and user-friendly tools exist for running various types of analyses (e.g., Qiime, Mothur, Megan, IMG/VR, Anvi'o, Qiita, MetaVir), scientists lack comprehensive software for easily building scalable, searchable, online data repositories on which they can rely during their ongoing research.

**Results:**

metaXplor is a scalable, distributable, fully web-interfaced application for managing, sharing, and exploring metagenomic data. Being based on a flexible NoSQL data model, it has few constraints regarding dataset contents and thus proves useful for handling outputs from both shotgun and metabarcoding techniques. By supporting incremental data feeding and providing means to combine filters on all imported fields, it allows for exhaustive content browsing, as well as rapid narrowing to find specific records. The application also features various interactive data visualization tools, ways to query contents by BLASTing external sequences, and an integrated pipeline to enrich assignments with phylogenetic placements. The project home page provides the URL of a live instance allowing users to test the system on public data.

**Conclusion:**

metaXplor allows efficient management and exploration of metagenomic data. Its availability as a set of Docker containers, making it easy to deploy on academic servers, on the cloud, or even on personal computers, will facilitate its adoption.

## Findings

### Background

The capacity to obtain DNA or RNA sequences without isolating or cultivating microorganisms from a given host or environmental sample through metagenomic techniques has been cardinal for our current understanding of viral and microbial diversity [1, 2]. As the application of such techniques ascertained the ubiquity and immense diversity of microorganisms, it also led to a more holistic view of the functioning of life [3, 4]. This change in paradigm revolutionizes the way we understand ecological processes [5, 6], the emergence of disease [7, 8], or the functioning of the human body [9]. As a corollary of the immense diversity of microorganisms, the use of high-throughput sequencing techniques associated with metagenomics results in the collection of huge amounts of molecular data. With the addition of new projects, the methodical storage and query of such heterogeneous data, including metabarcoding and shotgun data, become increasingly difficult and stable tools that provide means to manage, share, and search them are required. While tools and platforms such as Qiime [10], Mothur [11], Megan [12], IMG/VR [13], Anvi’o [14], Qiita [15] or Metavir [16] offer extensive sets of tools to analyse and compare datasets obtained from distinct metagenomic projects, they are not specifically designed for the identification of distant homologies or tracking newly discovered sequence/gene families across projects. Thus, metaXplor was developed to ease the search of viral sequences within existing projects using similarity-based search algorithms and phylogenetic tools. Along with these sequence-centric functionalities, the platform also facilitates keeping track of study data and (re-)analysing them. We considered it useful to provide the community with a user-friendly online system to explore large sequence datasets and easily extract parts of them for later reuse.

### Application description

metaXplor is a sequence-centric web-interfaced application that is designed for managing, sharing, and exploring metagenomic datasets. Being distributable, its main features are to (i) centralize them at the laboratory or institute level, (ii) share them with local collaborators or partner scientists, (iii) easily filter on provided metadata to quickly get hold of sequences of interest at any time, (iv) compare external sequences with those contained in the system, and (v) refine provided taxonomic assignments using phylogenetic placement. The application is accessible via a web browser. It can handle multiple database hosts (defined via a configuration file), each of them being likely to point to several databases. An administration interface previously proven in Gigwa v2 [17] allows for managing databases, projects, users, and permissions. It provides means to manage data privacy levels, to suppress existing data, and to define which users can consult or amend existing datasets.

### Data import

Administrators can import project data themselves or grant users permission to do so. Imports can be achieved by supplying a zip archive (either by uploading it or by specifying its http URL) containing 4 types of files:

- A tab-delimited text file providing sample metadata, including 3 standard BioSample [18] attribute names (sample_name, collection_date, lat_lon) and any additional user-defined fields;
- A second tab-delimited text file, used for specifying how samples contributed to each sequence in the project: these numeric values may represent the number of reads from each sample that are recruited by a contig in the case of shotgun metagenomic data, or per sample operational taxonomic unit abundances in the case of metabarcoding data;
- A standard FASTA file providing nucleotide information for all sequences mentioned in the latter;
- A third tab-delimited text file providing assignment details for all sequences that were successfully assigned to NCBI accessions, also based on user-defined fields. For compatibility with various processing methods that may be used for generating data, several assignment lines may be provided for a single sequence, and/or several accession IDs may be supplied (as CSV) on each assignment line. A bash script for converting tabular BLAST outputs to the appropriate format can be downloaded from the documentation page.

Imported sequences are thus divided into 2 categories: assigned to known accessions or unassigned. For each imported project, nucleotide and protein BLAST [19] banks are automatically created using all associated sequences, in order to allow for subsequent query. The contents of all fields present in the sample and assignment files are stored and indexed in a NoSQL database. The system caches relationships between NCBI accessions and taxonomy IDs in order to link each assigned sequence to a taxon. Whenever necessary, the cache contents are enriched during import by invoking NCBI's Entrez [20] web services. If several accession IDs are supplied for a single assignment, then the first common ancestor of their taxa is added to the corresponding record.

### Data exploration

All assigned sequences present in the system are searchable via the exploration interface, which makes it possible to work simultaneously on any combination of projects from the selected database. Color codes are applied to sequence-level, sample-level, and assignment-level fields for quick identification. This versatile interface provides means to combine filters on any of the fields added via project imports. Various kinds of advanced filtering widgets are thus proposed depending on the field's data type:

- plain lists for text fields containing ≤1,000 distinct values;
- autocompleting lists for text fields containing >1,000 distinct values;
- minimum-maximum ranges for numeric and date fields;
- tree-based selector for the taxonomy field;
- visual geographic map selector for the sample collection location field, based on Leaflet [21], OpenStreetMap [22], and Carto [23] technology.

**Figure 1:**
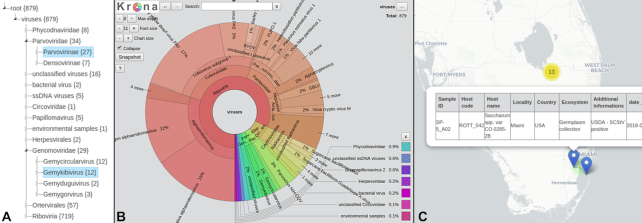
Graphical dataset representations: (A) taxonomic tree featuring per-taxon sequence counts; (B) Krona pie displaying the same data in a more interactive manner; (C) zoomable, draggable sample collection location map with icons linking to full sample information.

Search results can be browsed in 4 different ways. The default display is a sortable table with selectable fields supporting pagination, which can be configured to group results at the sequence, sample, or assignment level. Table rows are clickable and lead to a dialog box with all the information related to the selected record. The other 3 displays, all interactive, allow search results to be browsed as a taxonomic tree, a Krona [24] pie chart, and a zoomable geographic map showing sample collection locations (Fig. 1).

When multiple assignment methods are involved in selected projects, the user is invited to select one of them for the construction of taxonomy trees and pies. In such cases the assignment-method widget is also active by default in the exploration filters (so is the best-hit widget when sequences contain multiple assignments) because this is necessary for result counts to be identical between the table view and the taxonomy views.

### Data export and phylogenetic assignment

Once a dataset of interest has been selected, it can be downloaded in the same formats as supported for imports: a FASTA sequence file, and tab-delimited text files providing sample metadata, sequence composition, or assignment information. Data can also be exported in the popular BIOM [25] format, thus allowing easy manipulation of exported data in a variety of visualization or analysis tools such as Phinch [26] or Calypso [27]. Because this format enforces a precise and limited set of taxonomy ranks, sequence metadata are enriched with a field named “full_taxonomy" that can include ranks beyond those defined in the BIOM format, e.g., several ranks associated with virus classification. Exports are automatically compressed into zip archives and may be either directed to the client computer for direct download or temporarily materialized as physical files on the web server. In the latter case, a download URL is provided, making it easy to share with collaborators or feed into external systems. Indeed, next to the export button, a “sharing” icon provides means to configure “online output tools” to which metaXplor will be able to push exported data. As an example, this feature is compatible with Galaxy [28] data sources and thus allows any exported file to be transferred into a Galaxy history by a simple button click. The metaXplor instance administrator can configure ≤5 default output tools, and each user can define a custom one for his personal purpose. This feature will facilitate conducting online analyses from selected datasets.

**Figure 2:**
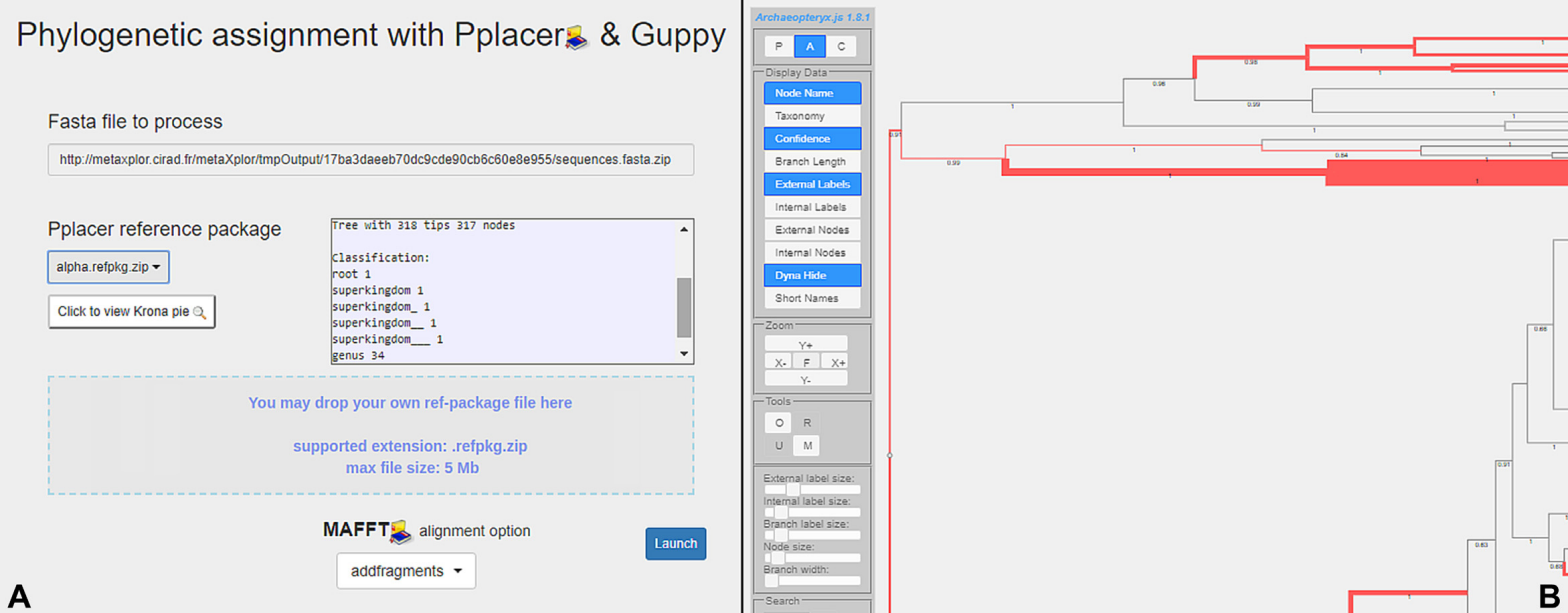
Phylogenetic assignment interface: (A) submission form allowing placement of exported or external sequences on an online or external reference tree, supporting add, addlong, and addfragments MAFFT alignment options; (B) Archeopteryx.js-driven interactive result display.

When a FASTA file is exported to the web server, the application offers to run a phylogenetic assignment on its contents. The user is then invited to either select a reference package [29] among those provided by the system (*de novo* generated or obtained from paprica [30]) or upload a custom refpkg archive. A nucleotide sequence alignment is first applied using MAFFT [31] v7.313 before pplacer [32] v1.1.alpha19 proceeds with positioning exported sequences onto the existing reference tree. Then, guppy [32] v1.1.alpha19 is used for sequence classification (“classify" option) and to generate an XML version of the pplacer tree (“fat" option). Last, Archeopteryx.js [33] is invoked to display an interactive solution for the end-user to investigate the results. After classification is performed, users with write permissions on any involved project have the facility to save newly found assignments to the database, thus enriching its contents for the benefit of all users. Figure 2 illustrates the user-friendliness of the phylogenetic placement feature.

### Running BLAST or Diamond against database contents

**Figure 3:**
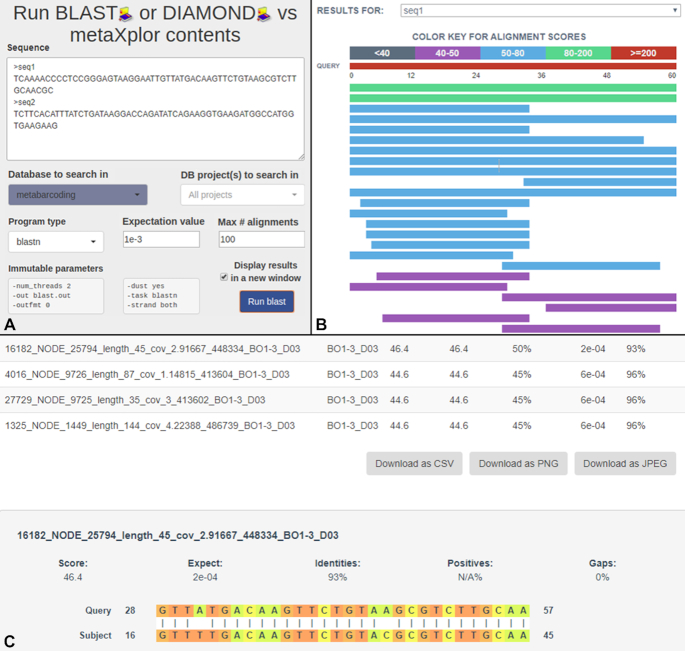
BLAST/Diamond functionality interface: (A) submission form allowing application of a selected search algorithm on multiple queries and subject projects, with adjustable evalue and num_alignments parameters; (B) BlasterJS-driven dynamic multiple query result view; (C) download options and alignment details, also handled by BlasterJS.

Another section in the application provides means to search for similarity between an external set of sequences and those present in the system, the latter being used as a reference bank. Available algorithms are BLAST v2.6.0 and Diamond [34] v2.0.4. Job results consist of a standard BLAST output file per selected target project, which can be investigated online in an interactive manner thanks to the BlasterJS [35] library, as shown in Fig. 3. Matching sequences can also be downloaded in FASTA format for further analyses (e.g., alignment, viral genome reconstruction).

Several BLAST types are supported: BLASTx (comparison of a DNA query sequence, after its translation into the 6 possible frames, with a protein sequence database) with Diamond as a faster alternative, BLASTp (comparison of a protein query sequence with a protein sequence database) with Diamond as a faster alternative, BLASTn (comparison of a DNA query sequence with a DNA sequence database), tBLASTn (comparison of a protein query with a DNA database, in the 6 possible frames of the database), and tBLASTx (comparison of the 6-frame translations of a nucleotide query sequence with the 6-frame translations of a nucleotide sequence database). This functionality was designed to provide means to quickly check whether newly obtained, locally held sequences share similarity with material already stored in previous projects.

## Architecture and Data Model

### Application architecture outline

**Figure 4:**
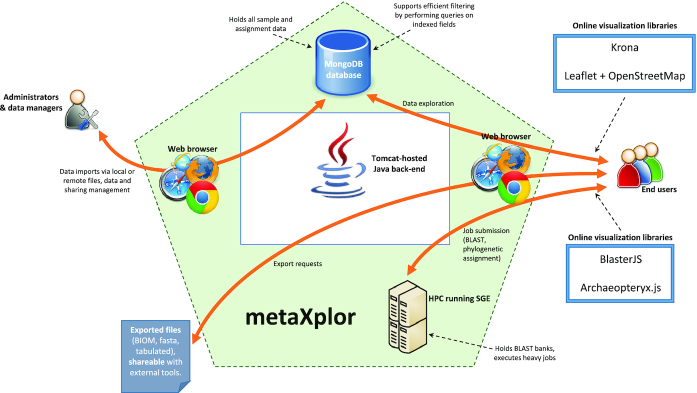
High-level diagram of metaXplor application illustrating its components and the interactions they establish between one another, and with users or administrators.

The software architecture of metaXplor (Fig. 4) can be described as follows:

- A standard HTML/Bootstrap/jQuery [36, 37] interface allows users and administrators to conveniently interact with the system;
- One or several MongoDB [38] servers are used as a persistence layer for data that are searchable via the “Explore” interface, i.e., all data except actual nucleotide sequences. Because MongoDB is a scalable solution that provides means to index >60 fields per collection, fast response times can be ensured even when running highly combined queries on large amounts of data;
- A high-performance computing (HPC) server running Oracle/Sun Grid Engine (SGE) [39] holds nucleotide and protein BLAST banks for each set of sequences involved in a project. This entity is responsible for running all CPU-intensive jobs except those that are database-related: BLAST/Diamond bank creation and query, phylogenetic assignment;
- A Java back-end consisting in a web application based on Spring Framework [40] acts as a central point for metaXplor and orchestrates data flow by interpreting user input, building database queries and sending them to MongoDB, invoking SGE via Opal Toolkit [41] web services, building GUI views and contents, compiling export files, and so forth. This component also keeps an indexed fasta file per project to allow quick access to nucleotide sequences when browsing/exporting data.

For metaXplor design, we focused on durability, maintainability, and extendibility by electing an industry development paradigm based on proven open-source standards such as the Spring Framework and Apache Tomcat. Regarding database needs, NoSQL seemed to be the best-suited solution for handling large datasets, and, more precisely, the MongoDB choice was found relevant because of its robustness, scalability, and schemaless design, which proved helpful in supporting user-defined fields. To our eyes, its large developer community also gives it status as a standard.

To ease deployment, the system is made available as a set of Docker [42] containers:

- MongoDB container: unmodified official Docker image for MongoDB document databases, which provides high availability and easy scalability. It is maintained by the Docker Community;
- HPC container: based on the official Docker image for Apache Tomcat, it embeds all tools required for detaching CPU-intensive jobs from the main web application. Thus, it features additional software such as SGE for job management (via an integration based on the docker-sge Dockerfile [43]), Opal Toolkit for interfacing with the latter, and all above-mentioned bioinformatics programs;
- Web application container: also based on the official Docker image for Apache Tomcat, it features the main metaXplor web application (Java back-end, HTML/Javascript interface).

This solution offers much flexibility in the sense that metaXplor can be straightforwardly configured in accordance with available hardware, from a minimal set-up on a workstation for testing purposes to a production environment where each container would run on a machine optimized for its purpose.

### Data model

**Figure 5:**
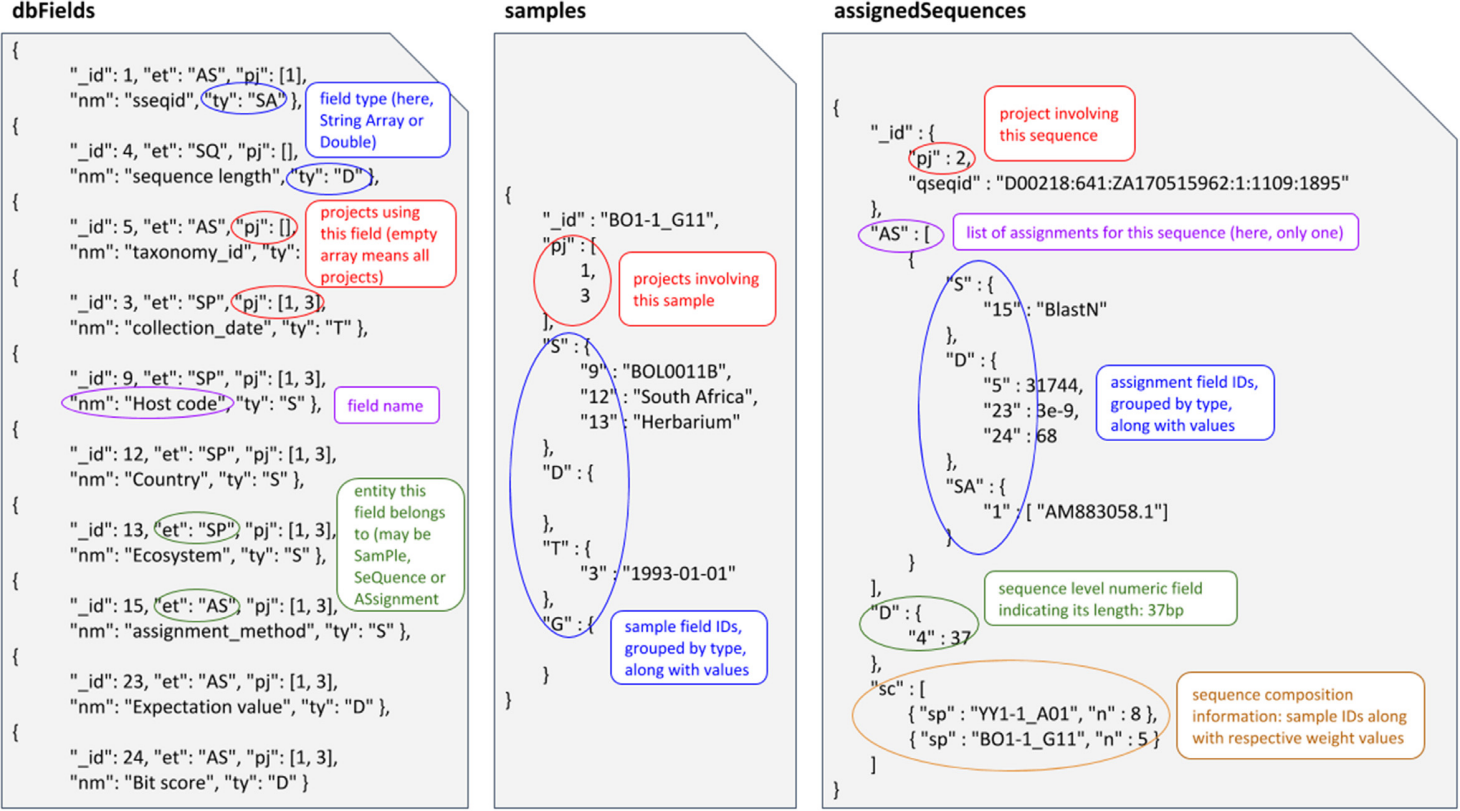
Sample contents of MongoDB collections holding searchable data: the dbFields collection holds the description of each searchable field (i.e., metadata) by storing the entity type (sequence, sample, or assignment) it describes, the list of projects it appears in, its verbose name, and its data type; the samples collection contains the list of projects in which each of them appears and all the sample metadata field values; the assignedSequences collection manages all assigned sequences by keeping track of their length along with sample contribution levels and the list of related assignments holding metadata field values.

In metaXplor, structured data (examples of the content of collections involved in the exploration functionality being given in Fig. 5) are organized in MongoDB as follows:

- A single “commons" database per instance contains collections holding reference data shared by all projects: NCBI taxonomy, accession-to-taxon mapping cache (described below), and reference package descriptions;
- Each metagenomic database added via the system consists of the following collections: projects (with attributes specified at import time), dbFields (list of dynamically added fields according to import file contents), samples, sequences (unassigned), assignedSequences (embedding assignments), and various cache collections (1 for BLAST results, 1 for phylogenetic assignment results, 1 for taxonomic trees, and 1 for each searchable field).

The central model entity in metaXplor's database structure is the “sequence." Each one originates from ≥1 “samples," as defined in the sequence composition file. For instance, a singleton read sequence would originate from only 1 sample whereas contigs may have been assembled using reads from various samples. Operational taxonomic unit representatives would also relate to the various samples in which they were detected.

Each sequence comes with ≥0 “assignments." Those that have none are stored separately as unassigned sequences and can only be BLASTed against, but not searched via the exploration interface. For those linked to several assignments, the presence of a best_hit flag per assignment method is required for 1 of these assignments. This is then taken into account when exporting in the BIOM format, and, as mentioned before, for building taxonomic trees or pies, which require a single taxon to be associated with each sequence.

Imported assignments may be directly provided with a taxonomy_id field. If not, they are required to be linked to ≥1 NCBI accession IDs. When a single ID is provided, the system attaches its corresponding taxon to the assignment record. In the case of multiple accession IDs, which typically occurs with metabarcoding data, the first common ancestor of their associated taxa is selected.

To efficiently perform this mapping task while supporting large data imports, the following mechanism was designed:

- The taxonomy associated to each of our ∼860,000 cached accession numbers is taken from SILVA-curated [44] when available, otherwise from NCBI's taxonomy database;
- Accession-to-taxonomy associations are stored as a cache that is first consulted when assignment records are imported;
- For accessions not found in the cache, their details are pulled from Entrez web services and added to it;
- Finally, assignment records are persisted with accession and taxon information. Web service invocation failures lead to storing no taxon ID; such records are detected later on by the system and new attempts to retrieve the missing information are performed.

Note that the accession cache collection lies in the shared “commons" database. This implies that when importing project data from a given user, any records added to the cache will not need to be retrieved from web services, should it be encountered again within the same application instance.

## Conclusions

metaXplor is a user-friendly, distributable web-interfaced data-repository that provides tools to easily combine and filter project metadata, spatial, and taxonomic information from multiple meta-omic projects (i.e., shotgun metagenomics, metabarcoding, metatranscriptomics). In addition to offering taxonomic assignment browsing, it provides a fully integrated pipeline to enrich assignments with phylogenetic placements. This unified interface will greatly help researchers apprehend the relation of a given set of sequences with those from the already known diversity. Additionally, metaXplor provides functionality to BLAST external sequences against those contained in its featured projects. Because a large fraction of sequences obtained from metagenomic projects remain unclassified, i.e., the so-called dark matter [45], referring to sequences not having any detectable similarity with existing classified sequences, this functionality provides means to confront both classified and unclassified sequences from distinct projects. Finally, as an open-source, web-oriented multi-user platform, the system is adapted for collaborative work and data sharing as illustrated by the possibility to push exported data into external tools such as Galaxy. Thus, at a time when making scientific data FAIR (Findable, Accessible, Interoperable, and Reusable) is becoming a priority, we believe that metaXplor will prove useful in many ways. In future versions, we will consider adding support for further visualization/analysis features, and facilitating communication with additional external tools.

## Availability and Requirements

 

## Data Availability

metaXplor's source code is available in the South Green GitHub repository [46]. Deployment can be achieved directly using the docker-compose.yml file it features, which automatically pulls required container images from Docker Hub [47]. Snapshots of our code and other supporting data are openly available in the *GigaScience* repository, GigaDB [48].

## Abbreviations

BLAST: Basic Local Alignment Search Tool; CPU: central processing unit; CSV: comma-separated values; GUI: graphical user interface; HPC: high-performance computing; MAFFT: Multiple Alignment using Fast Fourier Transform; NCBI: National Center for Biotechnology Information.

## Competing Interests

The authors declare that they have no competing interests.

## Funding

This work was supported by the Agropolis Foundation grant E-SPACE (1504–004).

## Authors’ Contributions

D.F. provided the original idea and sample viral shotgun metagenomic datasets, followed development closely, tested the system, and reported bugs. G.S. designed the application structure and data model. G.B. implemented the initial version of the dynamic advanced filtering widgets. A.P. and G.S. wrote the applicative code, fixed bugs, and implemented most of the GUI. A.P. integrated most HPC-powered tools. P.L. designed and tested the phylogenetic assignment functionality and provided reference packages for it. P.R. helped put the team together and provided funding for internships and travel. F.M. provided expertise in handling metabarcoding data. M.A. created the Docker containers and finalized and optimized some of the import code. G.S. and P.L. wrote the manuscript. All authors read and approved the final version of the manuscript.

## Supplementary Material

giab001_GIGA-D-20-00212_Original_Submission

giab001_GIGA-D-20-00212_Revision_1

giab001_GIGA-D-20-00212_Revision_2

giab001_Response_to_Reviewer_Comments_Original_Submission

giab001_Response_to_Reviewer_Comments_Revision_1

giab001_Reviewer_1_Report_Original_Submission Christopher Hunter, Ph.D. -- 7/30/2020 Reviewed

giab001_Reviewer_1_Report_Revision_1 Christopher Hunter, Ph.D -- 11/30/2020 Reviewed

giab001_Reviewer_2_Report_Original_Submission Gregory Vey, Ph.D. -- 8/8/2020 Reviewed

giab001_Reviewer_3_Report_Original_Submission Justine Debelius -- 8/11/2020 Reviewed

giab001_Reviewer_3_Report_Revision_1 Justine Debelius -- 11/26/2020 Reviewed
