# Supplementary material for: metaXplor: an interactive viral and microbial metagenomic data manager
Source: Gigascience. 2021 Feb 2;10(2):giab001. doi: 10.1093/gigascience/giab001 (PMC7931823; doi:10.1093/gigascience/giab001)

GigaScience

metaXplor: an interactive viral and microbial metagenomic data manager

--Manuscript Draft--

Manuscript Number:	GIGA-D-20-00212R2
Full Title:	metaXplor: an interactive viral and microbial metagenomic data manager
Article Type:	Technical Note
Funding Information:	Agropolis Fondation (1504-004) — Not applicable
Abstract:	Background<br><br>Efficiently managing large, heterogeneous data in a structured yet flexible way is a challenge to research laboratories working with genomic data. Specifically regarding both shotgun- and metabarcoding-based metagenomics, while online reference databases and user-friendly tools exist for running various types of analyses (e.g. Qiime [1], Mothur [2], Megan [3], MetaVir [4], IMG/VR [5], Anvi'o [6]), scientists lack comprehensive software for easily building scalable, searchable, online data repositories that they may rely on during their ongoing research.<br><br>Results<br><br>metaXplor is a scalable, distributable, fully web-interfaced application for managing, sharing and exploring metagenomic data. Being based on a flexible NoSQL data model, it has very few constraints regarding dataset contents, and thus proves useful for handling outputs from both shot-gun and metabarcoding techniques. By supporting incremental data feeding and providing means to combine filters on all imported fields, it allows for exhaustive content browsing, as well as rapid narrowing to find very specific records. The application also features various interactive data visualization tools, ways to query contents by BLASTing external sequences, and an integrated pipeline to enrich assignments with phylogenetic placements. The project home page provides the URL of a live instance allowing users to test the system on public data.<br><br>Conclusion<br><br>metaXplor allows efficient management and exploration of metagenomic data. Its availability as a set of Docker containers, making it easy to deploy on academic servers, on the cloud or even on personal computers, will undoubtedly facilitate its adoption.
Corresponding Author:	Guilhem Sempéré<br>CIRAD<br>Montpellier Cedex 5, FRANCE
Corresponding Author Secondary Information:	
Corresponding Author's Institution:	CIRAD
Corresponding Author's Secondary Institution:	
First Author:	Guilhem Sempéré
First Author Secondary Information:	
Order of Authors:	Guilhem Sempéré<br>Adrien Pételৎ<br>Magsen Abbé<br>Pierre Lefeuvre<br>Philippe Roumagnac

Powered by Editorial Manager® and ProduXion Manager® from Aries Systems Corporation

	Frédéric Mahé
	Gaël Baurens
	Denis Filloux
**Order of Authors Secondary Information:**	
**Response to Reviewers:**	Dear editor,<br><br>Please find below our point-by-point response to all remarks issued by the reviewers.<br><br><br>Reviewer #1:<br><br>REMARK: on https://metaxplor.cirad.fr/metaXplor/main.jsp I clicked "Explore data", then "search" without changing any of the values/dropdowns just as it appears by default. Then I clicked the Taxonomy tree view icon, when I selected "BlastX" from the dropdown menu a dialog box popped onto the screen saying just "metaxplor.cirad.fr says" with nothing else, and no content to the page. I'm guessing that there are no blastx results to be displayed, but the dialog box doesn't say anything?! When I change the dropdown back to BlastN it all works fine again.<br>REPLY: Thanks for identifying that bug, it has been fixed on the live instance and applied to the latest Docker images<br><br><br>Reviewer #3:<br><br>REMARK: citing qiita is not suffecient to explain how this database is functionally different<br>REPLY: Thanks for the comment. After presenting alternative tools (Qiita and Metavir), we now clearly specified at the bottom the Findings' section's Background paragraph that these tools "are not specifically designed for the identification of distant homologies or tracking newly discovered sequence/gene families". Conversely, these functionalities are at the root of metaXplor's design, making it "sequence-centric" in opposition to the more "project centric" functionalities of Qiita and Metavir.<br><br>REMARK: Different analyses, yes, but how do I get biological insight from a largely descriptive krona plot and a map? What does the pplacer buy me over export into something like iTOL?<br>REPLY: The interest in running pplacer from within metaXplor is that it saves exporting / reimporting into another tool, and that it allows to enrich database contents with newly found assignments (both facts are now mentioned in the manuscript)<br><br><br>Hoping that these modifications will satisfy your requests, we remain available for further instructions.<br><br>Best regards,<br><br>Guilhem Sempéré
**Additional Information:**	
**Question**	**Response**
Are you submitting this manuscript to a special series or article collection?	No
**Experimental design and statistics**<br><br><br>Full details of the experimental design and statistical methods used should be given in the Methods section, as detailed in our	Yes

Minimum Standards Reporting Checklist. Information essential to interpreting the data presented should be made available in the figure legends.<br><br>Have you included all the information requested in your manuscript?	
**Resources**<br><br>A description of all resources used, including antibodies, cell lines, animals and software tools, with enough information to allow them to be uniquely identified, should be included in the Methods section. Authors are strongly encouraged to cite Research Resource Identifiers (RRIDs) for antibodies, model organisms and tools, where possible.<br><br>Have you included the information requested as detailed in our Minimum Standards Reporting Checklist?	Yes
**Availability of data and materials**<br><br>All datasets and code on which the conclusions of the paper rely must be either included in your submission or deposited in publicly available repositories (where available and ethically appropriate), referencing such data using a unique identifier in the references and in the "Availability of Data and Materials" section of your manuscript.<br><br>Have you have met the above requirement as detailed in our Minimum Standards Reporting Checklist?	Yes

Manuscript with DOI

Click here to access/download;Manuscript;metaXplor_manuscript_1220_rev

metaXplor: an interactive viral and microbial metagenomic data manager

Sempéré G[1,2,5], Pétel A[3], Abbé M[1,5], Lefeuvre P[3], Roumagnac P[4,6], Mahé F[4,6], Baurens G[1,5], Filloux D[4,6]

1. CIRAD, UMR INTERTRYP, F-34398 Montpellier, France
2. South Green Bioinformatics Platform, Bioversity, CIRAD, INRAE, IRD, Montpellier, France
3. CIRAD, UMR PVBMT, F-97410 St Pierre, La Réunion, France
4. CIRAD, BGPI, 34398 Montpellier, France
5. INTERTRYP, Univ Montpellier, CIRAD, IRD, Montpellier, France
6. BGPI, INRAE, CIRAD, Institut Agro, Univ Montpellier, 34398 Montpellier, France

Abstract

Background: Efficiently managing large, heterogeneous data in a structured yet flexible way is a challenge to research laboratories working with genomic data. Specifically regarding both shotgun- and metabarcoding-based metagenomics, while online reference databases and user-friendly tools exist for running various types of analyses (e.g. Qiime [1], Mothur [2], Megan [3], MetaVir [4], IMG/VR [5], Anvi'o [6]), scientists lack comprehensive software for easily building scalable, searchable, online data repositories that they may rely on during their ongoing research.

Results: metaXplor is a scalable, distributable, fully web-interfaced application for managing, sharing and exploring metagenomic data. Being based on a flexible NoSQL data model, it has very few constraints regarding dataset contents, and thus proves useful for handling outputs from both shot-gun and metabarcoding techniques. By supporting incremental data feeding and providing means to combine filters on all imported fields, it allows for exhaustive content browsing, as well as rapid narrowing to find very specific records. The application also features various interactive data visualization tools, ways to query contents by BLASTing external sequences, and an integrated pipeline to enrich assignments with phylogenetic placements. The project home page provides the URL of a live instance allowing users to test the system on public data.

Conclusion: metaXplor allows efficient management and exploration of metagenomic data. Its availability as a set of Docker containers, making it easy to deploy on academic servers, on the cloud or even on personal computers, will undoubtedly facilitate its adoption.

Keywords

Metagenomics; Data management; NoSQL; NGS; Sample; Sequence; Assignment; Taxonomy; Web; Shotgun; Metabarcoding

Findings

Background

The capacity to obtain DNA or RNA sequences without isolating or cultivating microorganisms from a given host or environmental sample through metagenomic techniques has been cardinal for our current understanding of viral and microbial diversity (Thomas et al., 2012 [7], Forbes et al., 2017 [8]). As the application of such techniques ascertained the ubiquity and immense diversity of microorganisms, it also led to a more holistic view of the functioning of life (Grice et Segre, 2012 [9], Stobbe et al., 2014 [10]). This change in paradigm revolutionizes the way we understand ecological processes (Coutinho et al., 2018 [11], Falkowski et al., 2008 [12]), the emergence of disease (Vayssier-Taussat et al., 2014 [13], Lefeuvre et al., 2019 [14]) or the functioning of the human body (The Human Microbiome Project Consortium, 2012 [15]). As a corollary of the immense diversity of microorganisms, the use of high throughput sequencing techniques associated with metagenomics results in the collection of huge amounts of molecular data. With the addition of new projects, the methodical storage and query of such heterogeneous data, including metabarcoding and shotgun data, become increasingly difficult and stable tools that provide means to manage, share and search them are required. While platforms such as Qiita [16] or Metavir [4] offer an extensive set of tools to analyse and compare datasets obtained from distinct metagenomic projects, they are not specifically designed for the identification of distant homologies or tracking newly discovered sequence/gene families across projects. Thus, metaXplor was developed to ease the search of viral sequences within existing projects using similarity based search algorithms and phylogenetic tools. Along with these sequence-centric functionalities, the platform also facilitates keeping track of study data and (re-)analyzing it. We considered it useful to provide the community with a user-friendly online system to explore large sequence datasets and easily extract parts of them for later reuse.

Application description

metaXplor is a sequence-centric web-interfaced application that is designed for managing, sharing and exploring metagenomic datasets. Being distributable, its main features are to (i) centralize them at the laboratory or institute level, (ii) share them with local collaborators or partner scientists, (iii) easily filter on provided metadata to quickly get hold of sequences of interest at any time, (iv) compare external sequences with those contained in the system, (v) refine provided taxonomic assignments using phylogenetic placement. The application is accessible via a web browser. It can handle multiple database hosts (defined via a configuration file), each of them being likely to point to several databases. An administration interface previously proven in Gigwa v2 [17] allows for managing databases, projects, users and permissions. It provides means to manage data privacy levels, to suppress existing data, and to define which users may consult or amend existing datasets.

Data import

Administrators may import project data themselves, or grant users permission to do so. Imports may be achieved by supplying a zip archive (either by uploading it or by specifying its http URL) containing four types of files:

- A tab-delimited text file providing sample metadata, including three standard BioSample [18] attributes names (sample_name, collection_date, lat_lon) and any additional user-defined fields;
- A second tab-delimited text file, used for specifying how samples contributed to each sequence in the project: these numeric values may represent the number of reads from each sample that are

recruited by a contig in the case of shotgun metagenomic data, or per sample OTU abundances in the case of metabarcoding data;

- A standard FASTA file providing nucleotide information for all sequences mentioned in the latter;
- A third tab-delimited text file providing assignment details for all sequences that were successfully assigned to NCBI accessions, also based on user-defined fields. For compatibility with various processing methods that may be used for generating data, several assignment lines may be provided for a single sequence, and/or several accession IDs may be supplied (as CSV) on each assignment line. A bash script for converting tabular BLAST outputs to the appropriate format may be downloaded from the documentation page.

Imported sequences are thus divided into two categories: assigned to known accessions, or unassigned. For each imported project, nucleotide and protein BLAST [19] banks are automatically created using all associated sequences, in order to allow for subsequent query. The contents of all fields present in the sample and assignment files are stored and indexed in a NoSQL database. The system caches relationships between NCBI accessions and taxonomy IDs in order to link each assigned sequence to a taxon. Whenever necessary, the cache contents are enriched during import by invoking NCBI's Entrez [20] web-services. If several accession IDs are supplied for a single assignment, then the first common ancestor of their taxa is added to the corresponding record.

Data exploration

All assigned sequences present in the system are searchable via the exploration interface, that allows to work simultaneously on any combination of projects from the selected database. Color codes are applied to sequence-level, sample-level and assignment-level fields for quick identification. This versatile interface provides means to combine filters on any of the fields added via project imports. Various kinds of advanced filtering widgets are thus proposed depending on the field's data type:

- plain lists for text fields containing up to 1000 distinct values;
- autocompleting lists for text fields containing more than 1000 distinct values;
- minimum-maximum ranges for numeric and date fields;
- tree-based selector for the taxonomy field;
- visual geographic map selector for the sample collection location field, based on Leaflet [21], OpenStreetMap [22] and Carto [23] technology.

Search results can be browsed in four different ways. The default display is a sortable table with selectable fields supporting pagination, which can be configured to group results at the sequence, sample or assignment level. Table rows are clickable and lead to a dialog box with all the information related to the selected record. The other three displays, all interactive, allow browsing search results as a taxonomic tree, a Krona [24] pie chart, and a zoomable geographic map showing sample collection locations (Fig. 1).

Figure 1:

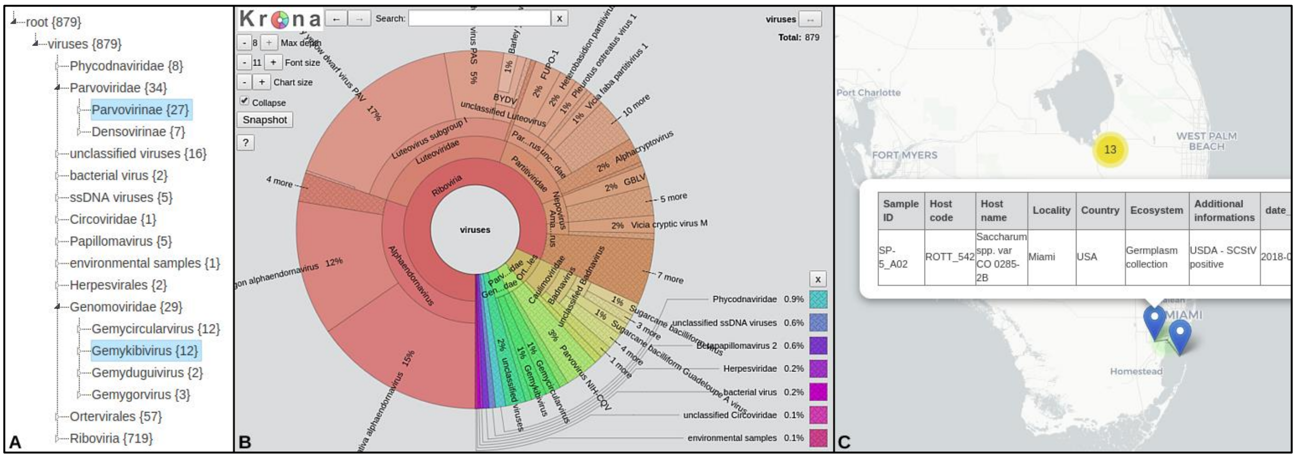

Graphical dataset representations: (A) taxonomic tree featuring per-taxon sequence counts; (B) Krona pie displaying the same data in a more interactive manner; (C) Zoomable, draggable sample collection location map with icons linking to full sample information.

When multiple assignment-methods are involved in selected projects, the user is invited to select one of them for the construction of taxonomy trees and pies. In such cases the assignment-method widget is also active by default in the exploration filters (so is the best-hit widget when sequences contain multiple assignments), as this is necessary for result counts to be identical between the table view and the taxonomy views.

Data export and phylogenetic assignment

Once a dataset of interest has been selected, it may be downloaded in the same formats as supported for imports: a FASTA sequence file, and tab-delimited text files providing sample metadata, sequence composition or assignment information. Data may also be exported in the popular BIOM [25] format, thus allowing easy manipulation of exported data in a variety of visualization or analysis tools such as Phinch [26] or Calypso [27]. Because this format enforces a precise and limited set of taxonomy ranks, sequence metadata are enriched with a *full_taxonomy* field that may include ranks beyond those defined in the BIOM format, e.g., several ranks associated with virus classification. Exports are automatically compressed into zip archives and may be either directed to the client computer for direct download, or temporarily materialized as physical files on the web server. In the latter case, a download URL is provided, making it easy to share with collaborators or feed into external systems. Indeed, next to the export button, a "sharing" icon provides means to configure "online output tools" that metaXplor will be able to push exported data to. As an example, this feature is compatible with Galaxy [28] data sources and thus allows to transfer any exported file into a Galaxy history, by a simple button click. The metaXplor instance administrator may configure up to five default output tools, and each user may define a custom one for his personal purpose. This feature will facilitate conducting online analyses from selected datasets.

When a FASTA file is exported to the web server, the application offers to run a phylogenetic assignment on its contents. The user is then invited to either select a reference package [29] among those provided by the system (*de novo* generated or obtained from paprica [30]), or upload a custom refpkg archive. A nucleotide sequence alignment is first applied using mafft [31] v7.313 before pplacer [32] v1.1.alpha19 proceeds with positioning exported sequences onto the existing reference tree. Then, guppy [32] v1.1.alpha19 is used for sequence classification (*classify* option) and to generate an XML version of the pplacer tree (*fat* option). Last, Archeopteryx.js [33] is invoked to display an interactive solution for the end-user to investigate the

results. After classification is performed, users with *write* permissions on any involved project have the facility to save newly found assignments to the database, thus enriching its contents for the benefit of all users. Fig. 2 illustrates the user-friendliness of the phylogenetic placement feature.

Figure 2:

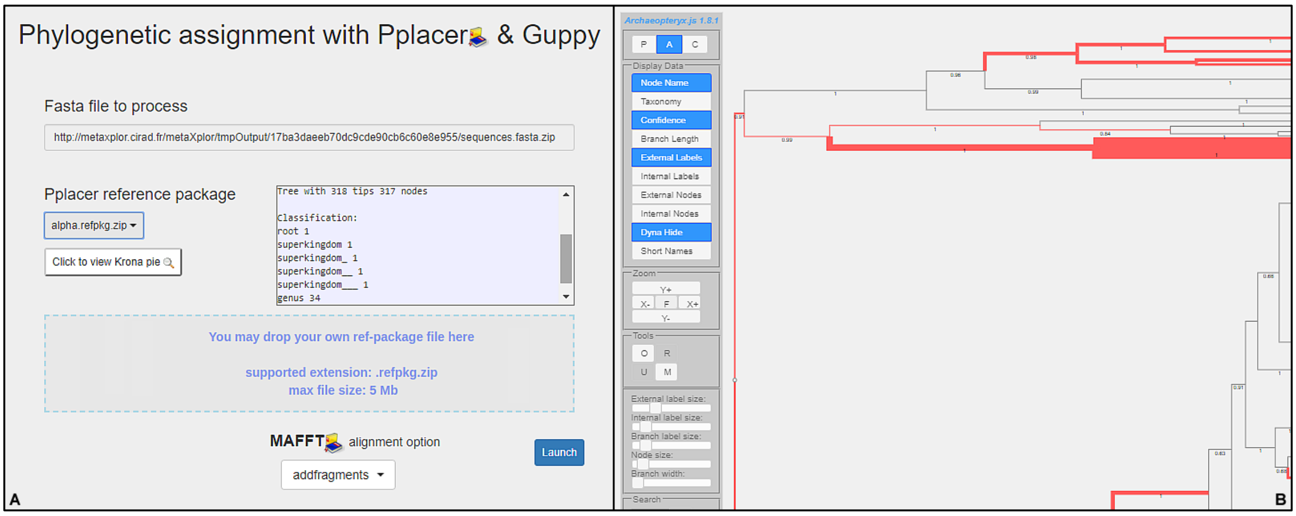

Phylogenetic assignment interface: (A) submission form allowing to place exported or external sequences on an online or external reference tree, supporting add, addlong and addfragments MAFFT alignment options; (B) Archeopteryx.js-driven interactive result display.

Running BLAST or Diamond against database contents

Another section in the application provides means to search for similarity between an external set of sequences and those present in the system, the latter being used as a reference bank. Available algorithms are BLAST v2.6.0 and Diamond [34] v2.0.4. Job results consist of a standard BLAST output file per selected target project, which may be investigated online in an interactive manner thanks to the BlasterJS [35] library, as shown in Fig. 3. Matching sequences may also be downloaded in FASTA format for further analyses (e.g. alignment, viral genome reconstruction).

Figure 3:

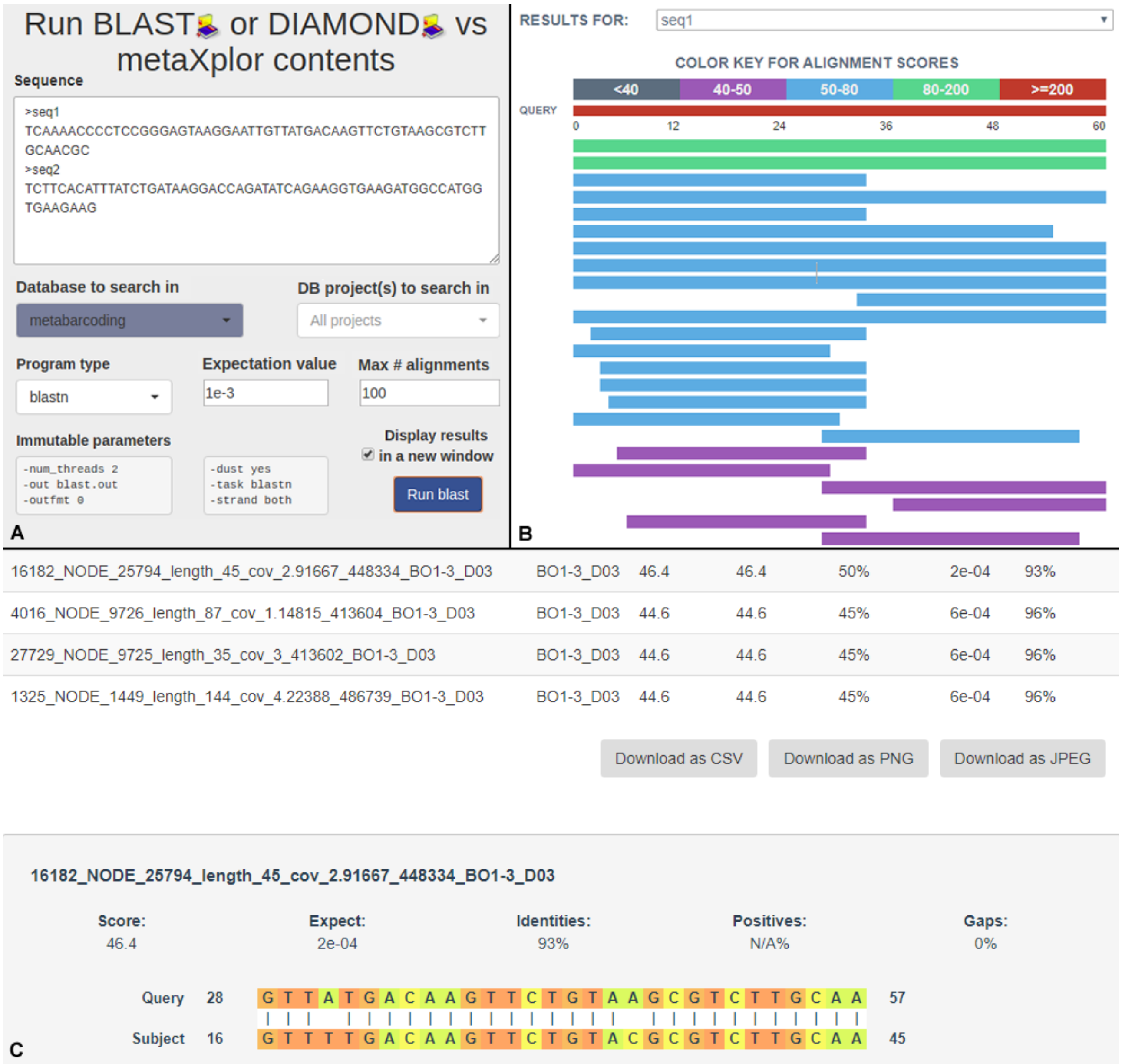

BLAST / Diamond functionality interface: (A) submission form allowing to apply a selected search algorithm on multiple queries and subject projects, with adjustable evalue and num_alignments parameters, (B) BlasterJS-driven dynamic multiple query result view, (C) download options and alignment details, also handled by BlasterJS.

Several BLAST types are supported: BLASTx (comparison of a DNA query sequence, after its translation into the 6 possible frames, with a protein sequence database) with Diamond as a faster alternative, BLASTp (comparison of a protein query sequence with a protein sequence database) with Diamond as a faster alternative, BLASTn (comparison of a DNA query sequence with a DNA sequence database), tBLASTn (comparison of a protein query with a DNA database, in the 6 possible frames of the database), tBLASTx (comparison of the six-frame translations of a nucleotide query sequence with the six-frame translations of a nucleotide sequence database). This functionality was designed to provide means to quickly check whether newly obtained, locally held sequences share similarity with material already stored in previous projects.

Architecture and data model

Application architecture outline

The software architecture of metaXplor (Figure 4) can be described as follows:

- A standard HTML / Bootstrap [36] / jQuery [37] interface allows users and administrators to conveniently interact with the system;
- One or several MongoDB [38] servers are used as a persistence layer for data that are searchable via the "Explore" interface, i.e., all data except actual nucleotide sequences. As MongoDB is a scalable solution that provides means to index over 60 fields per collection, fast response times can be ensured even when running highly combined queries on large amounts of data;
- An HPC server running Oracle/Sun Grid Engine (SGE) [39] holds nucleotide and protein BLAST banks for each set of sequences involved in a project. This entity is responsible for running all CPU-intensive jobs except those that are database-related: BLAST / Diamond bank creation and query, phylogenetic assignment;
- A Java back-end consisting in a web application based on Spring Framework [40] acts as a central point for metaXplor, and orchestrates data flow by interpreting user input, building database queries and sending them to MongoDB, invoking SGE via Opal Toolkit [41] web-services, building GUI views and contents, compiling export files, etc. This component also keeps an indexed fasta file per project to allow quick access to nucleotide sequences when browsing / exporting data.

Figure 4:

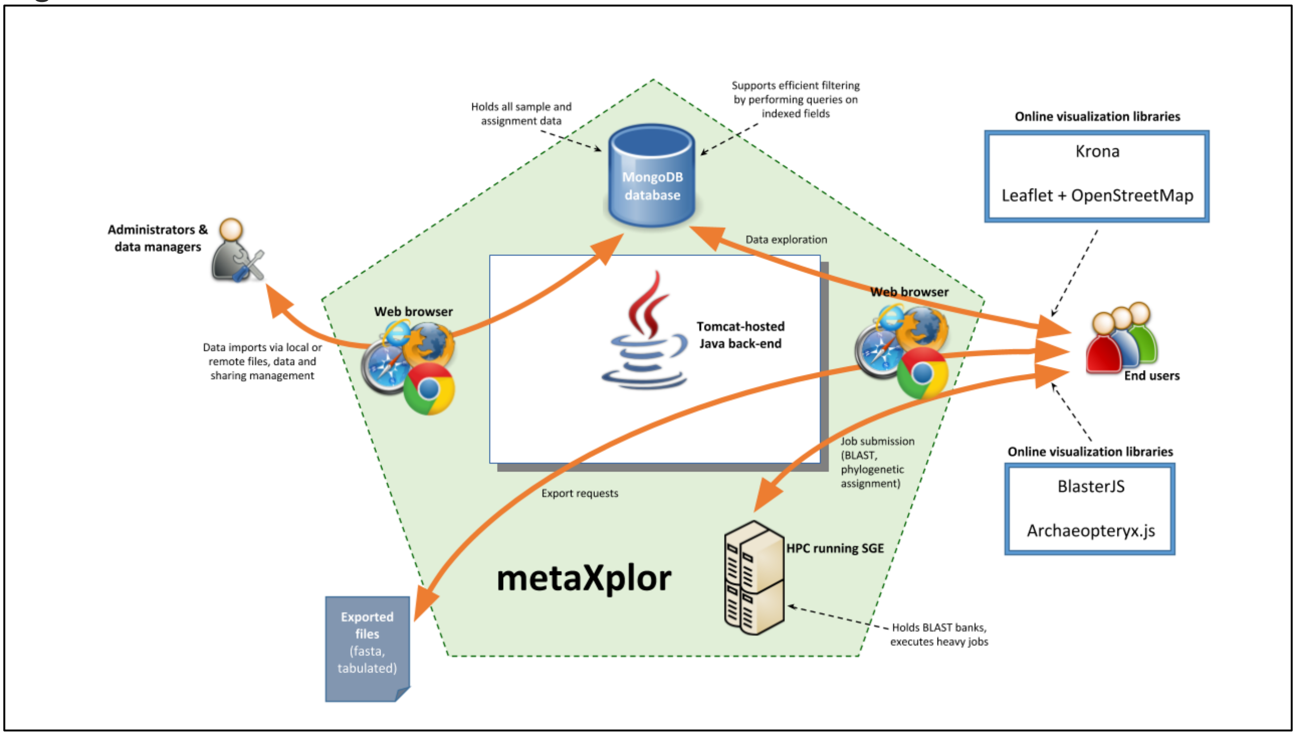

metaXplor application's high-level diagram illustrating its components and the interactions they establish between one another, and with users or administrators.

For metaXplor design, we focused on durability, maintainability, and extendibility by electing an industry development paradigm based on proven open-source standards such as the Spring Framework and Apache Tomcat. Regarding database needs, NoSQL appeared as the best-suited solution for handling large datasets, and, more precisely, the MongoDB choice was found relevant because of its robustness,

scalability, and schemaless design which proved very helpful in supporting user-defined fields. To our eyes, its large developer community also makes it stand as a standard.

To ease deployment, the system is made available as a set of Docker [42] containers:

- MongoDB container: unmodified official Docker image for MongoDB document databases which provides high availability and easy scalability. It is maintained by the Docker Community;

- HPC container: based on the official Docker image for Apache Tomcat, it embeds all tools required for detaching CPU-intensive jobs from the main web application. Thus, it features additional software such as SGE for job management (via an integration based on the docker-sge Dockerfile [43]), Opal Toolkit for interfacing with the latter, and all above-mentioned bioinformatics programs;

- Web application container: also based on the official Docker image for Apache Tomcat, it features the main metaXplor web application (Java backend, HTML / Javascript interface).

This solution offers much flexibility in the sense that metaXplor may be straightforwardly configured in accordance with available hardware, from a minimal setup on a workstation for testing purposes, to a production environment where each container would run on a machine optimized for its purpose.

Data model

In metaXplor, structured data (examples of the content of collections involved in the exploration functionality being given in Figure 5) are organized in MongoDB as follows:

- A single *commons* database per instance contains collections holding reference data shared by all projects: NCBI taxonomy, accession-to-taxon mapping cache (described below), and reference package descriptions;

- Each metagenomic database added via the system consists of the following collections: projects (with attributes specified at import time), dbFields (list of dynamically added fields according to import file contents), samples, sequences (unassigned), assignedSequences (embedding assignments), and various cache collections (one for BLAST results, one for phylogenetic assignment results, one for taxonomic trees, and one for each searchable field).

Figure 5:

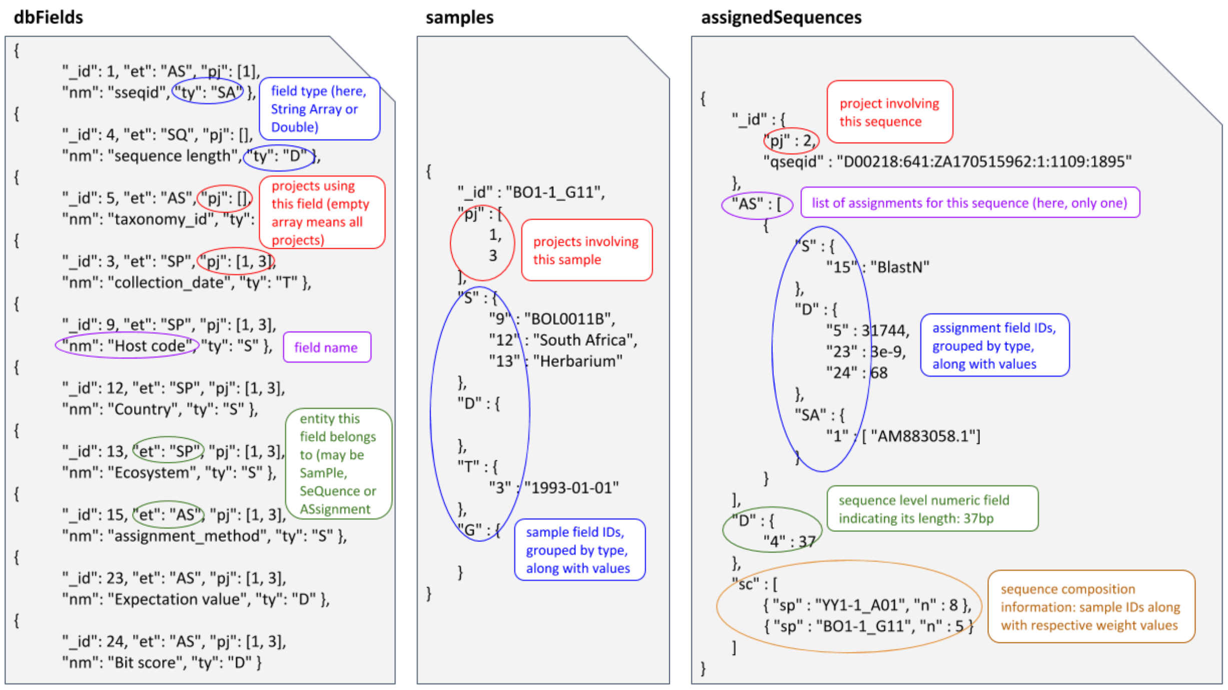

Sample contents of MongoDB collections holding searchable data: the dbFields collection holds the description of each searchable field (i.e., metadata) by storing the entity type (sequence, sample or assignment) it describes, the list of projects it appears in, its verbose name and its data type; the samples collection contains the list of projects each of them appears in and all the sample metadata field values; the assignedSequences collection manages all assigned sequences by keeping track of their length along with sample contribution levels and the list of related assignments holding metadata field values.

The central model entity in metaXplor's database structure is the **sequence**. Each one originates from *one or more* **samples**, as defined in the sequence composition file. For instance, a singleton read sequence would originate from only one sample whereas contigs may have been assembled using reads from various samples. OTU representatives would also relate to the various samples in which they were detected.

Each sequence comes with *zero or more* **assignments**. Those that have none are stored separately as unassigned sequences and may only be BLASTed against, but not searched via the exploration interface. For those linked to several assignments, the presence of a **best_hit** flag per assignment method is required for one of these assignments. This is then taken into account when exporting in the BIOM format, and, as mentioned before, for building taxonomic trees or pies, which require a single taxon to be associated with each sequence.

Imported assignments may be directly provided with a taxonomy_id field. If not, they are required to be linked to *one or more* NCBI **accession** IDs. When a single ID is provided, the system attaches its corresponding **taxon** to the assignment record. In the case of multiple accession IDs, which typically occurs with metabarcoding data, the first common ancestor of their associated taxa is selected.

In order to efficiently perform this mapping task while supporting large data imports, the following mechanism was designed:

- The taxonomy associated to each of our ~860,000 cached accession numbers is taken from SILVA-curated [44] when available, otherwise from NCBI's taxonomy database;
- Accession-to-taxonomy associations are stored as a cache which is first consulted when assignment records are imported;

- For accessions not found in the cache, their details are pulled from Entrez web-services, and added to it;
- Finally, assignment records are persisted with accession and taxon information. Web-service invocation failures lead to storing no taxon ID; such records are detected later on by the system and new attempts to retrieve the missing information are performed.

Note that the accession cache collection lies in the shared *commons* database. This implies that when importing project data from a given user, any records added to the cache will not need to be retrieved from web-services, should it be encountered again within the same application instance.

Conclusions

metaXplor is a user-friendly, distributable web-interfaced data-repository that provides tools to easily combine and filter project metadata, spatial and taxonomic information from multiple meta-omic projects (i.e., shotgun metagenomics, metabarcoding, metatranscriptomics). In addition to offering taxonomic assignment browsing, it provides a fully integrated pipeline to enrich assignments with phylogenetic placements. This unified interface will greatly help researchers apprehend the relation of a given set of sequences with those from the already known diversity. Additionally, metaXplor provides functionality to BLAST external sequences against those contained in its featured projects. Since a large fraction of sequences obtained from metagenomic projects remains unclassified, i.e. the so-called dark matter (Roux et al., 2015 [45]) referring to sequences not having any detectable similarity with existing classified sequences, this functionality provides means to confront both classified and unclassified sequences from distinct projects. Finally, as an open-source, web-oriented multi-user platform, the system is adapted for collaborative work and data sharing as illustrated by the possibility to push exported data into external tools such as Galaxy. Thus, at a time when making scientific data FAIR (Findable, Accessible, Interoperable, and Re-usable) is becoming a priority, we believe that metaXplor will prove useful in many ways. In future versions, we will consider adding support for further visualization / analysis features, and facilitating communication with additional external tools.

Availability and requirements

- Project name: metaXplor
- Project home page: https://github.com/SouthGreenPlatform/metaXplor
- Research Resource Identifier: metaXplor, RRID:SCR_019025
- Elixir bio.tools Identifier: biotools:metaxplor
- Operating system(s): Platform-independent
- Programming languages: Java, MongoDB, HTML, Javascript
- Requirements: Java v8 or higher, Tomcat v8.5 or higher, MongoDB v4.2 or higher, Docker v19 or higher, Docker-compose v1.17 or higher , an internet connection
- License: GNU Affero General Public License v3.0

- Restrictions to use for non-academics: None
- DockerHub repositories: https://hub.docker.com/r/guilhemsempere/metaxplor-webapp and https://hub.docker.com/r/guilhemsempere/metaxplor-hpc

Availability of Supporting Data

metaXplor's source code is available in the South Green GitHub repository [46]. Deployment may be achieved directly using the docker-compose.yml file it features, which automatically pulls required container images from Docker Hub [47]. Snapshots of our code and other supporting data are openly available in the GigaScience repository, GigaDB [48].

Declarations

Acknowledgements

The authors thank the South Green Platform [49] team for technical support. We are also grateful to Jean-Marc Mienville for careful reading that helped improve the manuscript, and express warm thanks to UMR AGAP and UMR BGPI for investing in a high-performance server used for hosting our metaXplor instance's database.

Authors' contributions

D.F. provided the original idea and sample viral shotgun metagenomic datasets, followed development closely, tested the system and reported bugs. G.S. designed the application structure and data model. G.B. implemented the initial version of the dynamic advanced filtering widgets. A.P. and G.S. wrote the applicative code, fixed bugs and implemented most of the GUI. A.P. integrated most HPC-powered tools. P.L. designed and tested the phylogenetic assignment functionality, and provided reference packages for it. P.R. helped put the team together and provided funding for internships and travel. F.M. provided expertise in handling metabarcoding data. M.A. created the Docker containers, finalized and optimized some of the import code. G.S. and P.L. wrote the manuscript. All contributors read and approved the final version of the manuscript.

Funding

This work was supported by the Agropolis Foundation grant E-SPACE (1504-004).

Competing interests

The authors declare that they have no competing interests.

Open Access

This article is distributed under the terms of the Creative Commons Attribution 4.0 International License (http://creativecommons.org/licenses/by/4.0/), which permits unrestricted use, distribution, and reproduction in any medium, provided users give appropriate credit to the original author(s) and source, provide a link to the Creative Commons license, and indicate if changes were made. The Creative Commons

Public Domain Dedication waiver (http://creativecommons.org/publicdomain/zero/1.0/) applies to the data made available in this article, unless otherwise stated.

References

1. Bolyen E, Rideout JR, Dillon MR, Bokulich NA, Abnet CC, Al-Ghalith GA, et al.. Reproducible, interactive, scalable and extensible microbiome data science using QIIME 2. *Nat Biotechnol*. 2019; doi: 10.1038/s41587-019-0209-9.

2. Schloss PD, Westcott SL, Ryabin T, Hall JR, Hartmann M, Hollister EB, et al.. Introducing mothur: Open-Source, Platform-Independent, Community-Supported Software for Describing and Comparing Microbial Communities. *Appl Environ Microbiol*. 2009; doi: 10.1128/AEM.01541-09.

3. Huson DH, Beier S, Flade I, Górska A, El-Hadidi M, Mitra S, et al.. MEGAN Community Edition - Interactive Exploration and Analysis of Large-Scale Microbiome Sequencing Data. Poisot T, editor. *PLOS Comput Biol*. 2016; doi: 10.1371/journal.pcbi.1004957.

4. Roux S, Tournayre J, Mahul A, Debroas D, Enault F. Metavir 2: new tools for viral metagenome comparison and assembled virome analysis. *BMC Bioinformatics*. 2014; doi: 10.1186/1471-2105-15-76.

5. Paez-Espino D, Chen I-MA, Palaniappan K, Ratner A, Chu K, Szeto E, et al.. IMG/VR: a database of cultured and uncultured DNA Viruses and retroviruses. *Nucleic Acids Res*. 2017; doi: 10.1093/nar/gkw1030.

6. Eren AM, Esen ÖC, Quince C, Vineis JH, Morrison HG, Sogin ML, et al.. Anvi'o: an advanced analysis and visualization platform for 'omics data. *PeerJ*. 2015; doi: 10.7717/peerj.1319.

7. Thomas T, Gilbert J, Meyer F. Metagenomics - a guide from sampling to data analysis. *Microb Inform Exp*. 2012; doi: 10.1186/2042-5783-2-3.

8. Forbes JD, Knox NC, Ronholm J, Pagotto F, Reimer A. Metagenomics: The Next Culture-Independent Game Changer. *Front Microbiol*. 2017; doi: 10.3389/fmicb.2017.01069.

9. Grice EA, Segre JA. The Human Microbiome: Our Second Genome. *Annu Rev Genomics Hum Genet*. Annual Reviews; 2012; doi: 10.1146/annurev-genom-090711-163814.

10. Stobbe AH, Roossinck MJ. Plant virus metagenomics: what we know and why we need to know more. *Front Plant Sci*. 2014; doi: 10.3389/fpls.2014.00150.

11. Coutinho FH, Gregoracci GB, Walter JM, Thompson CC, Thompson FL. Metagenomics Sheds Light on the Ecology of Marine Microbes and Their Viruses. *Trends Microbiol*. 2018; doi: 10.1016/j.tim.2018.05.015.

12. Falkowski PG, Fenchel T, Delong EF. The microbial engines that drive Earth's biogeochemical cycles. *Science*. 2008; doi: 10.1126/science.1153213.

13. Vayssier-Taussat M, Albina E, Citti C, Cosson J-F, Jacques M-A, Lebrun M-H, et al.. Shifting the paradigm from pathogens to pathobiome: new concepts in the light of meta-omics. *Front Cell Infect Microbiol*. 2014; doi: 10.3389/fcimb.2014.00029.

14. Lefeuvre P, Martin DP, Elena SF, Shepherd DN, Roumagnac P, Varsani A. Evolution and ecology of plant viruses. *Nat Rev Microbiol*. 2019; doi: 10.1038/s41579-019-0232-3.

15. The Human Microbiome Project Consortium. Structure, function and diversity of the healthy human microbiome. *Nature*. 2012; doi: 10.1038/nature11234.

16. Gonzalez A, Navas-Molina JA, Kosciolek T, McDonald D, Vázquez-Baeza Y, Ackermann G, et al.. Qiita: rapid, web-enabled microbiome meta-analysis. *Nat Methods*. 2018; doi: 10.1038/s41592-018-0141-9.

17. Sempéré G, Pétel A, Rouard M, Frouin J, Hueber Y, De Bellis F, et al.. Gigwa v2—Extended and improved genotype investigator. *GigaScience*. 2019; doi: 10.1093/gigascience/giz051.

18. : BioProject and BioSample databases at NCBI: facilitating capture and organization of metadata | Nucleic Acids Research | Oxford Academic. https://academic.oup.com/nar/article/40/D1/D57/2903284 Accessed 2020 Nov 13.

19. Altschul SF, Gish W, Miller W, Myers EW, Lipman DJ. Basic local alignment search tool. *J Mol Biol*. 1990; doi: 10.1016/S0022-2836(05)80360-2.

20. Gibney G, Baxevanis AD. Searching NCBI Databases Using Entrez. *Curr Protoc Hum Genet*. 2011; doi: 10.1002/0471142905.hg0610s71.

21. : Leaflet - a JavaScript library for interactive maps. https://leafletjs.com/ Accessed 2020 May 5.

22. Haklay M, Weber P. OpenStreetMap: User-Generated Street Maps. *IEEE Pervasive Comput*. 2008; doi: 10.1109/MPRV.2008.80.

23. . CartoDB/CartoDB-basemaps. CARTO;

24. Ondov BD, Bergman NH, Phillippy AM. Interactive metagenomic visualization in a Web browser. *BMC Bioinformatics*. 2011; doi: 10.1186/1471-2105-12-385.

25. McDonald D, Clemente JC, Kuczynski J, Rideout JR, Stombaugh J, Wendel D, et al.. The Biological Observation Matrix (BIOM) format or: how I learned to stop worrying and love the ome-ome. *GigaScience*. 2012; doi: 10.1186/2047-217X-1-7.

26. Bik HM, Pitch Interactive. Phinch: An interactive, exploratory data visualization framework for –Omic datasets. Genomics; 2014 Oct.

27. Zakrzewski M, Proietti C, Ellis JJ, Hasan S, Brion M-J, Berger B, et al.. Calypso: a user-friendly web-server for mining and visualizing microbiome–environment interactions. *Bioinformatics*. 2016; doi: 10.1093/bioinformatics/btw725.

28. : Galaxy: A platform for interactive large-scale genome analysis. https://genome.cshlp.org/content/15/10/1451 Accessed 2020 Nov 13.

29. . fhcrc/taxtastic. FHCRC Computational Biology;

30. : Microbial Communities Can Be Described by Metabolic Structure: A General Framework and Application to a Seasonally Variable, Depth-Stratified Microbial Community from the Coastal West Antarctic Peninsula. https://journals.plos.org/plosone/article?id=10.1371/journal.pone.0135868 Accessed 2020 May 19.

31. Katoh K, Standley DM. MAFFT Multiple Sequence Alignment Software Version 7: Improvements in Performance and Usability. *Mol Biol Evol*. 2013; doi: 10.1093/molbev/mst010.

32. Matsen FA, Kodner RB, Armbrust EV. pplacer: linear time maximum-likelihood and Bayesian

phylogenetic placement of sequences onto a fixed reference tree. *BMC Bioinformatics*. 2010; doi: 10.1186/1471-2105-11-538.

33. : Archaeopteryx - cmzmasek. https://sites.google.com/site/cmzmasek/home/software/archaeopteryx (2019). Accessed 2019 Dec 30.

34. Buchfink B, Xie C, Huson DH. Fast and sensitive protein alignment using DIAMOND. *Nat Methods*. 2015; doi: 10.1038/nmeth.3176.

35. Blanco-Míguez A, Fdez-Riverola F, Sánchez B, Lourenço A. BlasterJS: A novel interactive JavaScript visualisation component for BLAST alignment results. *PLOS ONE*. 2018; doi: 10.1371/journal.pone.0205286.

36. contributors MO Jacob Thornton, and Bootstrap: Bootstrap. https://getbootstrap.com/ (2019). Accessed 2019 Dec 30.

37. js.foundation JF-. jQuery.

38. : The most popular database for modern apps. MongoDB. https://www.mongodb.com Accessed 2020 Nov 13.

39. . Oracle Grid Engine. Wikipedia.

40. : spring.io. https://spring.io/ (2019). Accessed 2019 Dec 30.

41. Ren J, Williams N, Clementi L, Krishnan S, Li WW. Opal web services for biomedical applications. *Nucleic Acids Res*. 2010; doi: 10.1093/nar/gkq503.

42. : Enterprise Container Platform. Docker. https://www.docker.com/ (2020). Accessed 2020 Jan 1.

43. Moss S. gawbul/docker-sge.

44. Yilmaz P, Parfrey LW, Yarza P, Gerken J, Pruesse E, Quast C, et al.. The SILVA and “All-species Living Tree Project (LTP)” taxonomic frameworks. *Nucleic Acids Res*. 2014; doi: 10.1093/nar/gkt1209.

45. Roux S, Hallam SJ, Woyke T, Sullivan MB. Viral dark matter and virus-host interactions resolved from publicly available microbial genomes. *eLife*. 2015; doi: 10.7554/eLife.08490.

46. SouthGreenPlatform/metaXplor. South Green Bioinformatics platform;

47. Docker Hub - Container Image Library | Docker. https://www.docker.com/products/docker-hub Accessed 2020 May 6.

48. Sempéré G; Pétel A; Abbé M; Lefeuvre P; Roumagnac P; Mahé F; Baurens G; Filloux D: Supporting data for "metaXplor: an interactive viral and microbial metagenomic data manager" GigaScience Database. 2020. http://dx.doi.org/10.5524/100852

49. The South Green portal: a comprehensive resource for tropical and Mediterranean crop genomics. *Curr Plant Biol*. 2016; doi: 10.1016/j.cpb.2016.12.002.

Figure

Click here to access/download;Figure;fig1.png

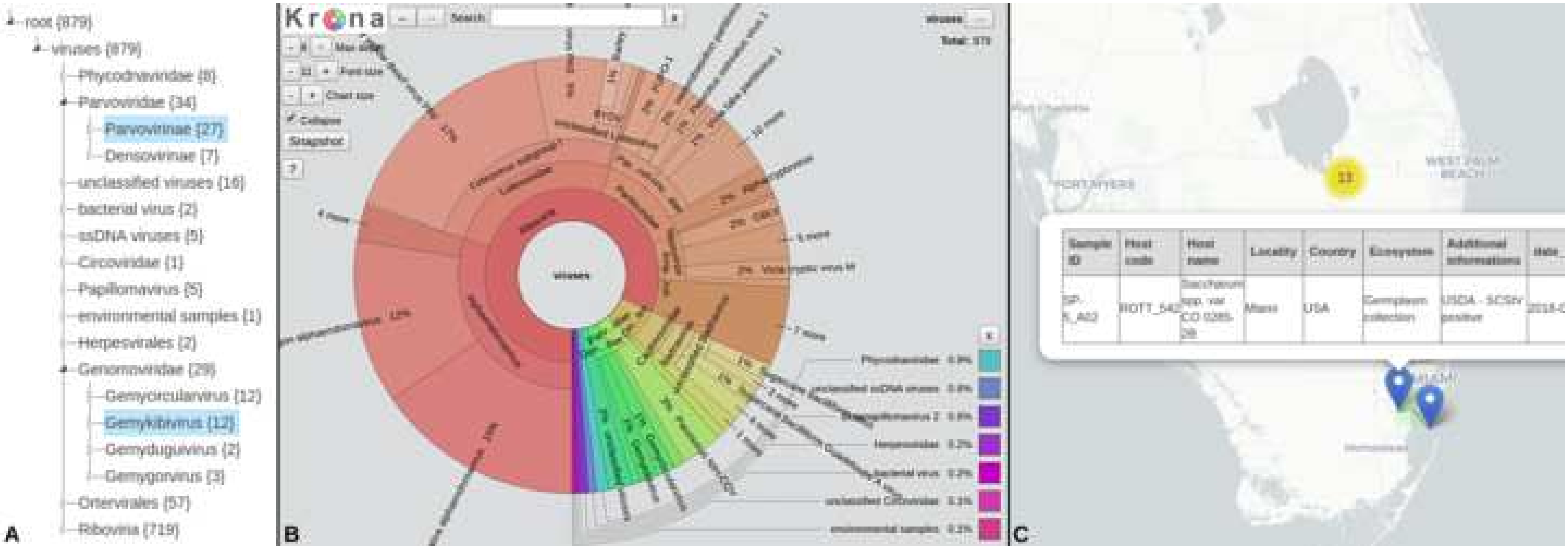

Figure

Click here to access/download;Figure;fig2.png

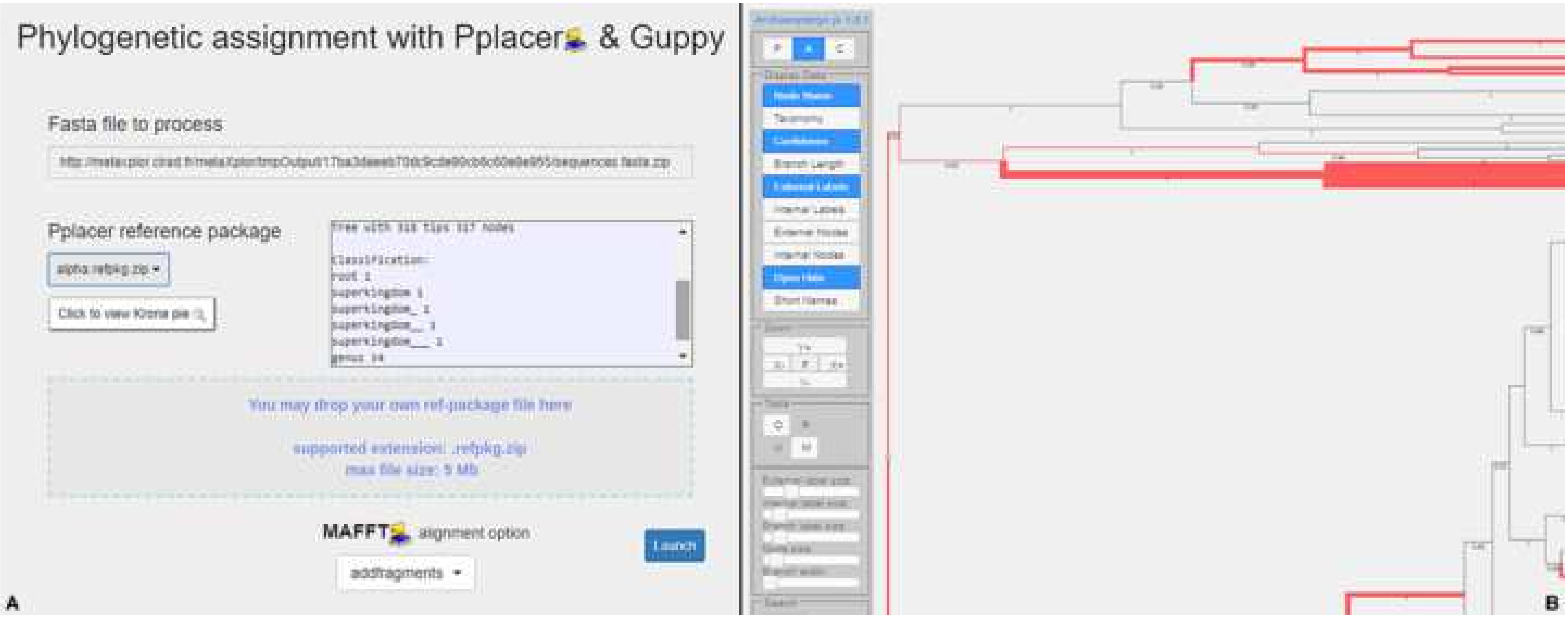

Figure

Click here to access/download;Figure;fig4.pptx

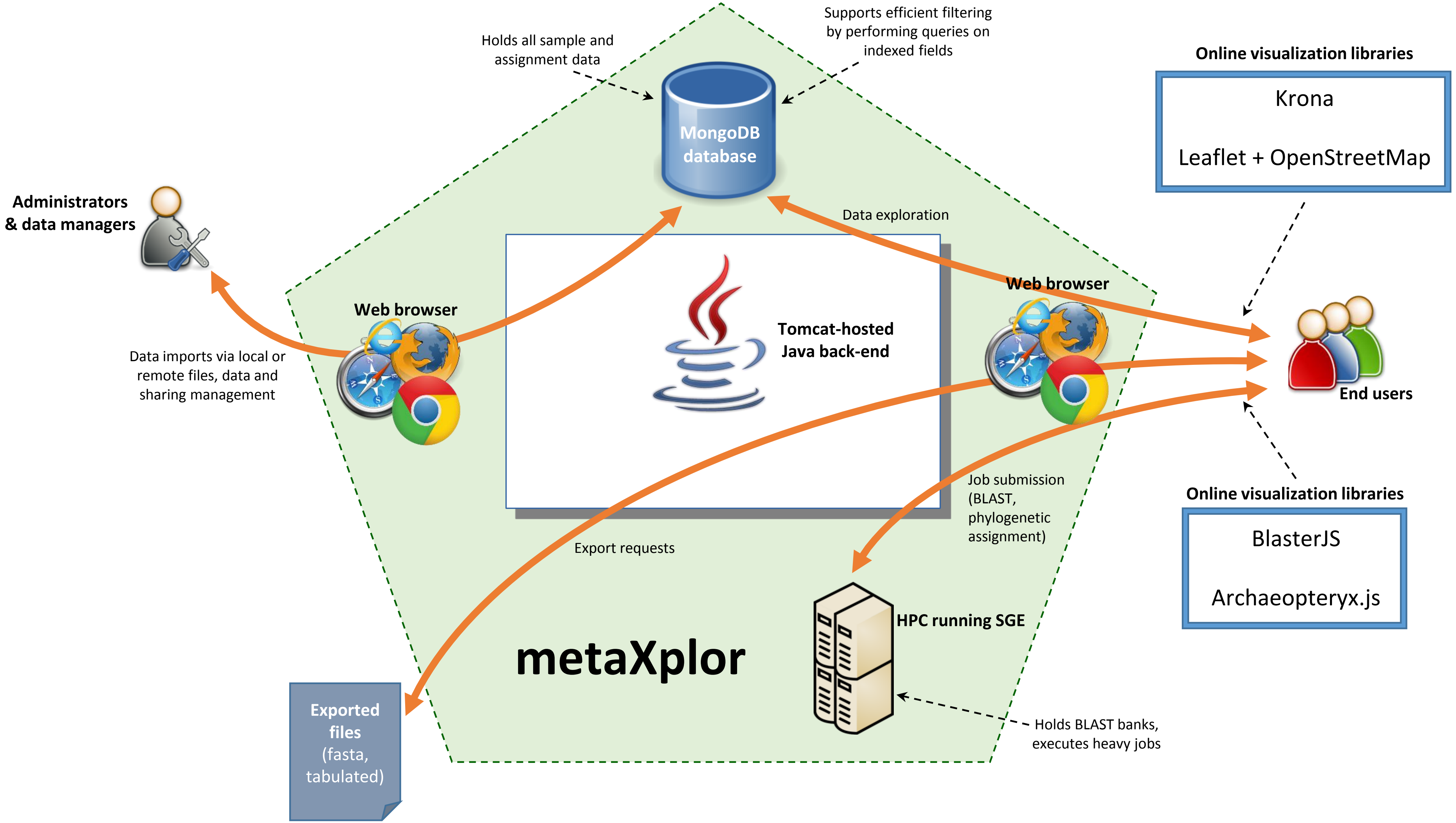

Figure Click here to access/download;Figure;fig3.png

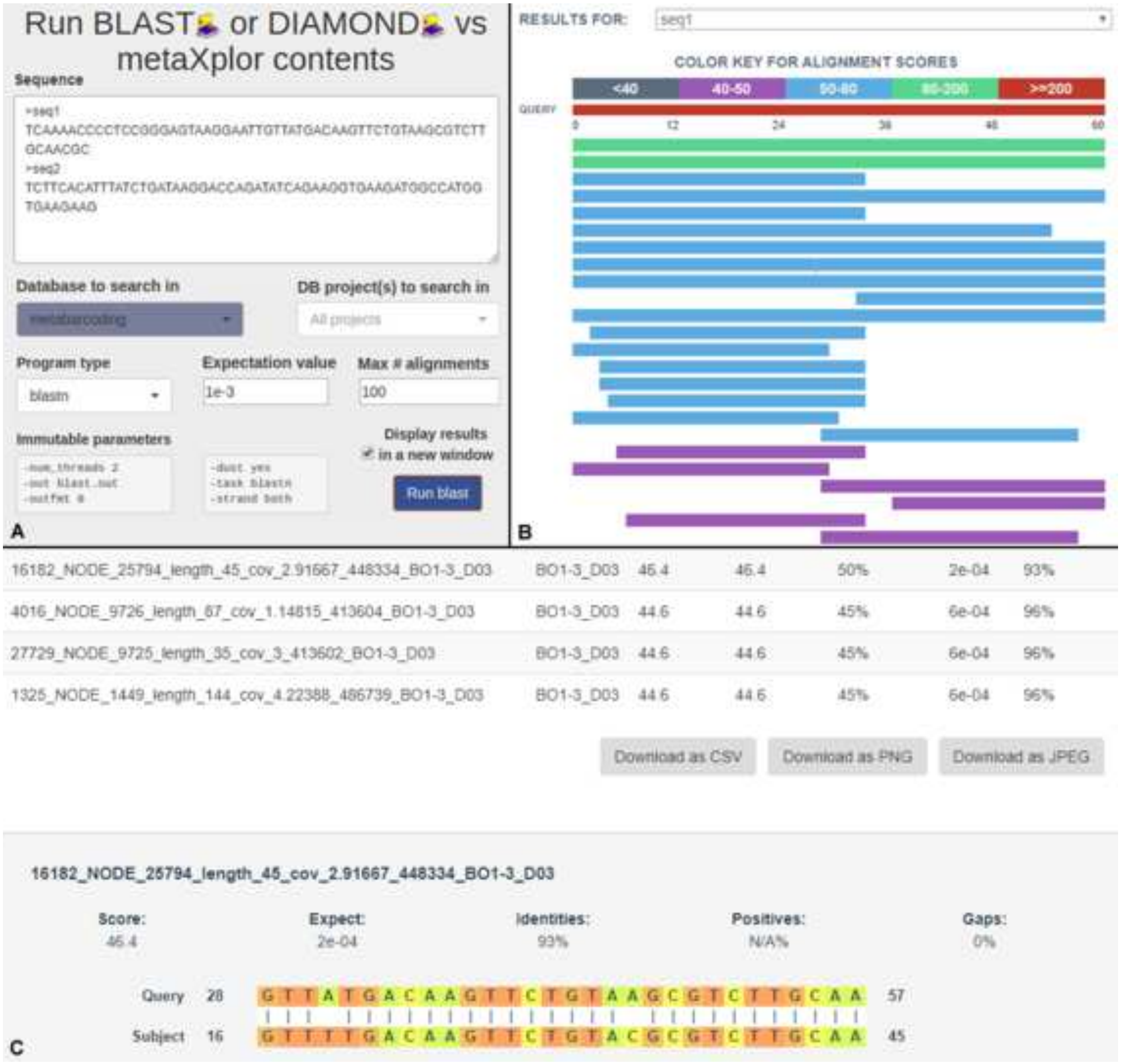

Click here to access/download;Figure;fig5.png

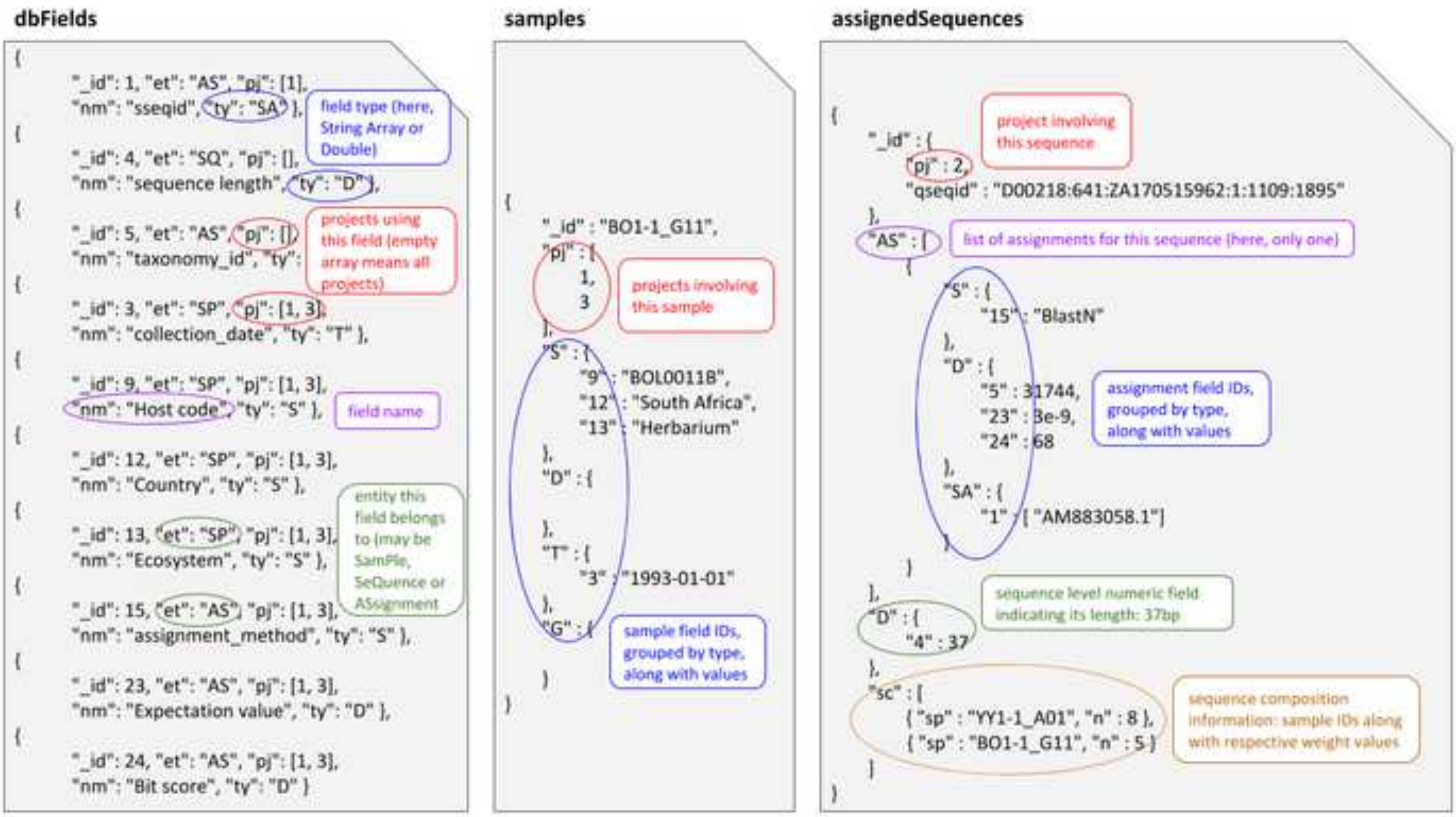

Supplement: giab001_GIGA-D-20-00212_Revision_2 [file giab001_giga-d-20-00212_revision_2.pdf]
