# Supplementary material for: metaXplor: an interactive viral and microbial metagenomic data manager
Source: Gigascience. 2021 Feb 2;10(2):giab001. doi: 10.1093/gigascience/giab001 (PMC7931823; doi:10.1093/gigascience/giab001)
Supplement: giab001_Response_to_Reviewer_Comments_Original_Submission [file giab001_response_to_reviewer_comments_original_submission.pdf]

Author's Response To Reviewer Comments

Close

Dear Editor and Reviewers,

Many thanks for the time spent on our manuscript and for the globally positive feedback you gave us. We tried our best to improve the application according to the review. A new version of the article is now available that takes into account changes applied to the software itself and tries to address as closely as possible the remarks you made. Please find below our replies to the comments and suggestions you submitted.

EDITOR

> please register any new software application in the bio.tools and SciCrunch.org databases to receive RRID (Research Resource Identification Initiative ID) and biotoolsID identifiers, and include these in your manuscript
> This is now done and stated in the “Availability and requirements” section:
Research Resource Identifier: metaXplor, RRID:SCR_019025
Elixir bio.tools Identifier: biotools:metaxplor

REVIEWER #1

> In general the webapp requires more documentation and online assistance, e.g. descriptions of terms used. For example, within the admin page to upload a new dataset the text box titles are very brief and have no additional explanation as to what is expected in each box. What exactly does "Samples available? *" mean? is it asking about the BioSample accessions for the sequence data or is it meaning the physical sample vouchers or something else?
> We added tooltips wherever possible, including in the data import form. We also significantly extended the online documentation by adding four sections that will help users better understand how to use the system.

> An opportunity seems to have been overlooked with respect to the sample metadata, while it is admirable that the system will accept any user specified attribute name this does make the ability to search and filter on specific terms more difficult. Perhaps there is scope to strongly encourage the use of attribute names that are recognised by INSDC or other authoritative bodies such as the Genomics Standards Consortium (GSC, - full disclosure, I am on the GSC board). By way of example; the three mandatory columns in the sample.tsv file (sample, gps_position, date_collect) are not consistent with terms of the same meaning in the BioSamples database or GSC recommendations; sample alias, collection_date, geographic location (latitude and longitude) or just "lat_lon".
> We modified these three field names to match the ones you pointed to. We also added a highlighted notice in the import form and the documentation page to advise users to use such standard names as much as possible.

> The assignments.tsv is a mandatory input file, but its format appears to be unique to this tool. Or perhaps its a variant of BLAST output I've not seen before. Is there an existing method to parse blast output to this format? Would it be possible to include a parser for users to import blastx tabular output directly?
> We consider that the supported input format is very open, and do not think it would be feasible to support file formats generated by the many existing processing pipelines. However, we understand that BLAST is a very common tool for performing the assignment step. The compromise we opted for was thus to write a bash script that translates BLAST format 7 -"Hit Table (text)" in NCBI interface- outputs into the expected input format. This script is linked from the documentation page and may be used as

an example for different kinds of pipeline outputs.

> I am intrigued to know how the system would cope with the large datasets being generated, for example the Mouse gut gene catalog (GigaDB dataset DOI:10.5524/100114) with 2.6 million genes from 184 samples. Currently this dataset has not been subjected to comparison to the NCBI database to determine the best-hit NCBI accessions required by metaXplore, instead the authors of that dataset used other methods to determine taxonomic content and functionality.
> We considered importing this dataset into metaXplor, however it raises two main difficulties :
- the fact that the originally submitted version could not be fed directly with taxon IDs but inevitably required NCBI accession names ;
- the fact that metaXplor expects raw counts to express the sample-to-sequence relationship whereas the mentioned dataset does not proceed so.
We were happy to address the first problem / limitation by amending the data model and import procedure to accept a taxonomy_id value when sseqid is not provided (now mentioned in the documentation). Unfortunately we did not succeed in refactoring the Mouse gut data to revert to raw counts and therefore could not import it into our system. However, we are confident that our server would be able to handle this dataset because although our largest database (BGPI_MicroQuar) contains less assigned sequences (622,266 when including a private project you may not see) its total number of assignments is 3,550,635. Also, the number of involved samples is <200 for the Mouse gut dataset and close to 1,500 in BGPI_MicroQuar.

> There is a minor point that is worth noting somewhere in the manuscript; the table of results displays all matches to the filters regardless of "Assignment method" unless you specifically filter on that facet. However, the other views (phylogenetic tree or Krona) both automatically filter on assignment method and cannot display all methods together. I understand why they do this (different methods gives multiple results for some sequences and therefore makes numerical interpretation impossible) but the fact the tabular data displays all by default makes the transition between table and other views a little confusing, i.e. the numbers are immediately different and you have to workout why. A simple solution might be to flag the assignment methods as a default filter in
the tabular, with the option to display all, this way users are immediately alerted to this feature and its then expected when moving to the other views.
> We proceeded as suggested and :
- made the assignment method (and best hit where applicable) filters active by default in the exploration interface when several methods were used to generate the selected project(s)
- clarified this point in the documentation and the manuscript

> This feature utilises BLAST, it appears to work in the webtool provided and is a desirable feature for some. I would question how well this part of the webapp will scale with increased numbers of large datasets. It maybe necessary to enable admin users to restrict its use on certain datasets?
> The idea behind using a job manager like SGE is that the administrator can set a maximum number of concurrent jobs for metaXplor to launch, this number being meant to take hardware possibilities into account. Jobs are put in a queue and only run when a slot is available for them. However in order to let this feature run smoother, we additionally implemented Diamond searches as a significantly faster alternative to BLAST.

> The phylogenetic assignment tool appears to be a stand-alone tool that is not linked to the datasets available in the webapp, i.e. just clicking that button takes you to a page where you have to upload your own fasta file of sequences and select and appropriate reference tree.
> We added some text on the feature's form submission page, and in the documentation, explaining how to assign sequences found in the system.

> Perhaps offering the user (or admin users only) the ability to run on any of the uploaded datasets to enrich them with phylogenetic assignments would be a nice addition, along with more comprehensive documentation on how to use the tool for user defined datasets.
> The phylogenetic assignment tools allow a more thorough investigation of sequences relationships. Whereas it is indeed run in an additional step, the results of the phylogenetic classification can be written in the assignation table within the main project database. Unfortunately we do not think it would be feasible to let users launch an online phylogenetic placement on an entire project's data. The reason behind this is that this assignment pipeline starts with a mafft multiple alignment step, which can be pretty time and resource consuming. Especially if we were to use a global refpkg as reference dataset.

REVIEWER #2

> Generally, there is a lack of citation for the opening assertions and a rather arbitrary citation list for landmark works. In particular, the Background does not stipulate the domain-specific problem that this tool proposes to solve or improve.
> We added citations for the opening assertions and tried to clarify our purpose in developing metaXplor.

> A review of similar and/or competing resources, or the lack thereof, would help to better characterize the contribution of the present work and help to avoid potential criticism of a derivative connection to Gigwa v2.
> Five citations were added to the article’s Background section, including one mentioning the differences with the Qiita database. We do not understand how metaXplor could be considered to derive from Gigwa. Although it is based on the same technology, it addresses totally different problems by storing types of data that have little in common.

> I am impressed by the authors' decision to use an industry standard software development paradigm. I feel that it would further strengthen this work to briefly remark on the durability, maintainability, and extendibility of their implementation, as a consequence of having elected to use freely available and well-accepted standards, such as the Spring Framework and Apache Tomcat. In my opinion, this approach lends significant credibility to the application architecture and is a refreshing change in a research domain presently inundated by ad hoc python scripting.
> Many thanks for this positive remark. We added a paragraph highlighting these facts to the manuscript’s « Application architecture outline » section.

> I can appreciate the decision to use NoSQL and the Data Model is well illustrated in Figure 5. However, given the prevalence of relational database management systems, it is necessary to briefly explain and justify the choice to use NoSQL in the present work, which is assumed to be motivated by the need for a schemaless model.
> The newly written, previously mentioned paragraph also states this.

> A more explicit discussion of how this tool relates to FAIR standards, or even just interoperability in general, would benefit this work.
> In order to enhance metaXplor’s usefulness, we implemented an additional feature that provides means to push exported data into external online tools such as Galaxy, which may then be used for further online analyses. This feature is mentioned in the new manuscript version, and the fact that metaXplor can contribute to make data FAIR is now mentioned in the conclusion.

REVIEWER #3

> it is unclear whether the docker container requires an internet connection - i.e. does metaXplor require this oracle backend to function
> We would like to point out that Oracle/Sun Grid Engine is not a database software but a HPC job scheduling software. The Docker containers can communicate with one another without an internet connection, as long as they are on the same local network. They can even run on the same server. However your remark is relevant because the system queries NCBI services to feed its accession cache and find their relationships with taxonomy. Therefore we specified in the manuscript’s Requirements section that an internet connection is required.

> One of the major issues that remains unaddressed in the manuscript is how this compares to the qiita database and associated infrastructure - a comprehensive database built for storing and analyzing studies.
> We referred to Qiita in the article’s Background section and mentioned the major differences that distinguish both systems.

> I would also like to try the interface, which I assume is what most end users will use. Is it possible for the authors to provide an anonymous reviewer login to explore/test the system?
> We are surprised that you could not find the CIRAD online instance (https://metaxplor.cirad.fr/) highlighted on the project homepage (https://github.com/SouthGreenPlatform/metaXplor) which is itself mentioned in the original manuscript’s « Availability and requirements » section. Reviewer #1 contacted

us directly and was immediately provided with a user account and some sample data that allowed him to even test data imports. Please let us know if you would also like to do so.

Thanks again for your feedback and suggestions. Best regards,

Guilhem Sempéré

Close
