# Supplementary material for: metaXplor: an interactive viral and microbial metagenomic data manager
Source: Gigascience. 2021 Feb 2;10(2):giab001. doi: 10.1093/gigascience/giab001 (PMC7931823; doi:10.1093/gigascience/giab001)
Supplement: giab001_Response_to_Reviewer_Comments_Revision_1 [file giab001_response_to_reviewer_comments_revision_1.pdf]

Author's Response To Reviewer Comments

Close

Dear editor,

Please find below our point-by-point response to all remarks issued by the reviewers.

Reviewer #1:

REMARK: on https://metaxplor.cirad.fr/metaXplor/main.jsp I clicked "Explore data", then "search" without changing any of the values/dropdowns just as it appears by default. Then I clicked the Taxonomy tree view icon, when I selected "BlastX" from the dropdown menu a dialog box popped onto the screen saying just "metaxplor.cirad.fr says" with nothing else, and no content to the page. I'm guessing that there are no blastx results to be displayed, but the dialog box doesn't say anything?! When I change the dropdown back to BlastN it all works fine again.
REPLY: Thanks for identifying that bug, it has been fixed on the live instance and applied to the latest Docker images

Reviewer #3:

REMARK: citing qiita is not suffecient to explain how this database is functionally different
REPLY: Thanks for the comment. After presenting alternative tools (Qiita and Metavir), we now clearly specified at the bottom the Findings' section's Background paragraph that these tools "are not specifically designed for the identification of distant homologies or tracking newly discovered sequence/gene families". Conversely, these functionalities are at the root of metaXplor's design, making it "sequence-centric" in opposition to the more "project centric" functionalities of Qiita and Metavir.

REMARK: Different analyses, yes, but how do I get biological insight from a largely descriptive krona plot and a map? What does the pplacer buy me over export into something like iTOL?
REPLY: The interest in running pplacer from within metaXplor is that it saves exporting / reimporting into another tool, and that it allows to enrich database contents with newly found assignments (both facts are now mentioned in the manuscript)

Hoping that these modifications will satisfy your requests, we remain available for further instructions.

Best regards,

Guilhem Sempéré

Close
