# Supplementary material for: metaXplor: an interactive viral and microbial metagenomic data manager
Source: Gigascience. 2021 Feb 2;10(2):giab001. doi: 10.1093/gigascience/giab001 (PMC7931823; doi:10.1093/gigascience/giab001)
Supplement: giab001_Reviewer_1_Report_Original_Submission — Christopher Hunter, Ph.D. -- 7/30/2020 Reviewed [file giab001_reviewer_1_report_original_submission.pdf]

Reviewer Report

Title: metaXplor: an interactive viral and microbial metagenomic data manager

Version: Original Submission **Date:** 7/30/2020

Reviewer name: Christopher Hunter, Ph.D.

Reviewer Comments to Author:

The manuscript is well written with clear English. In general I think this has the potential to be a very useful tool for the community, and I am happy to recommend this manuscript for publication if the authors can address a number of minor revisions. In addition it should be noted that I was unable to install the software tool myself due to limitations in my skill set and the editors should seek the advise of a specialist reviewer to confirm the technical details and the description of installation processes are adequate.

Using the webtool instance provided by the authors (https://metaxplor.cirad.fr/metaXplor/main.jsp) I was able to explore the user interface as described in the manuscript.

In general the webapp requires more documentation and online assistance, e.g. descriptions of terms used. For example, within the admin page to upload a new dataset the text box titles are very brief and have no additional explanation as to what is expected in each box. What exactly does "Samples available? *" mean? is it asking about the BioSample accessions for the sequence data or is it meaning the physical sample vouchers or something else?

An opportunity seems to have been overlooked with respect to the sample metadata, while it is admirable that the system will accept any user specified attribute name this does make the ability to search and filter on specific terms more difficult. Perhaps there is scope to strongly encourage the use of attribute names that are recognised by INSDC or other authoritative bodies such as the Genomics Standards Consortium (GSC, - full disclosure, I am on the GSC board). By way of example; the three mandatory columns in the sample.tsv file (sample, gps_position, date_collect) are not consistent with terms of the same meaning in the BioSamples database or GSC recommendations; sample alias, collection_date, geographic location (latitude and longitude) or just "lat_lon".

I was able to upload a user-defined dataset to the tool using the example dataset kindly provided by the authors. However I failed to create my over dataset from existing data in GigaDB due to the lack of a suitably formatted "assignments.tsv" file.

The assignments.tsv is a mandatory input file, but its format appears to be unique to this tool. Or perhaps its a variant of BLAST output I've not seen before. Is there an existing method to parse blast output to this format? Would it be possible to include a parser for users to import blastx tabular output directly?

I am intrigued to know how the system would cope with the large datasets being generated, for example the Mouse gut gene catalog (GigaDB dataset DOI:10.5524/100114) with 2.6 million genes from 184 samples. Currently this dataset has not been subjected to comparison to the NCBI database to determine the best-hit NCBI accessions required by metaXplore, instead the authors of that dataset used other methods to determine taxonomic content and functionality.

The stated goal of the tool is best captured by the line "metaXplor is a web-interfaced application that is designed for managing, sharing and exploring metagenomic datasets." The authors list 5 features that I address here with my comments:
(i) centralize them at the laboratory or institute level.
- I am not able to assess this functionality as it requires greater informatics skills that I have, please ensure another reviewer checks this part.
(ii) share them with local collaborators or partner scientists.
- After being granted admin role in the authors instance of the webtool I was able to upload a dataset and can see how to share it with other registered users.
(iii) easily filter on provided metadata to quickly get hold of sequences of interest at any time
- This feature is extremely useful and could be of significant interest to users looking to identify specific gene hits across multiple datasets. It may also be useful to filter "hits" by bit score or e-value. There is a minor point that is worth noting somewhere in the manuscript; the table of results displays all matches to the filters regardless of "Assignment method" unless you specifically filter on that facet. However, the other views (phylogenetic tree or Krona) both automatically filter on assignment method and cannot display all methods together. I understand why they do this (different methods gives multiple results for some sequences and therefore makes numerical interpretation impossible) but the fact the tabular data displays all by default makes the transition between table and other views a little confusing, i.e. the numbers are immediately different and you have to workout why. A simple solution might be to flag the assignment methods as a default filter in the tabular, with the option to display all, this way users are immediately alerted to this feature and its then expected when moving to the other views.
(iv) compare external sequences with those contained in the system
- This feature utilises BLAST, it appears to work in the webtool provided and is a desirable feature for some. I would question how well this part of the webapp will scale with increased numbers of large datasets. It maybe necessary to enable admin users to restrict its use on certain datasets?
(v) refine provided taxonomic assignments using phylogenetic placement
I have tested this feature, the text in the manuscript provides enough instruction for a user to be able to carry out the process, but there is no documentation available within the tool to assist with this process. As a naÃ¯ve user I would not be able to perform this functionality as it is not intuitive. My initial attempt failed simple because I clicked the "phylogenetic assignment" button in the webtool rather than Export my selection. The phylogenetic assignment tool appears to be a stand-alone tool that is not linked to the datasets available in the webapp, i.e. just clicking that button takes you to a page where you have to upload your own fasta file of sequences and select and appropriate reference tree. Useful, but not directly apparent how it fits in with the metaXplore app. Perhaps offering the user (or admin users only) the ability to run on any of the uploaded datasets to enrich them with phylogenetic assignments would be a nice addition, along with more comprehensive documentation on how to use the tool for user defined datasets.
Overall the app is good and the average user should be able to workout most of the features but some additional documentation would be a very useful addition.

Level of Interest

Please indicate how interesting you found the manuscript: Choose an item.

Quality of Written English

Please indicate the quality of language in the manuscript: Choose an item.

Declaration of Competing Interests

Please complete a declaration of competing interests, considering the following questions:

- Have you in the past five years received reimbursements, fees, funding, or salary from an organisation that may in any way gain or lose financially from the publication of this manuscript, either now or in the future?
- Do you hold any stocks or shares in an organisation that may in any way gain or lose financially from the publication of this manuscript, either now or in the future?
- Do you hold or are you currently applying for any patents relating to the content of the manuscript?
- Have you received reimbursements, fees, funding, or salary from an organization that holds or has applied for patents relating to the content of the manuscript?
- Do you have any other financial competing interests?
- Do you have any non-financial competing interests in relation to this paper?

If you can answer no to all of the above, write 'I declare that I have no competing interests' below. If your reply is yes to any, please give details below.

I am a GigaScience employee. Additionally, I am a volunteer member of the Genomics Standards Consortium.

I agree to the open peer review policy of the journal. I understand that my name will be included on my report to the authors and, if the manuscript is accepted for publication, my named report including any attachments I upload will be posted on the website along with the authors' responses. I agree for my report to be made available under an Open Access Creative Commons CC-BY license (http://creativecommons.org/licenses/by/4.0/). I understand that any comments which I do not wish to be included in my named report can be included as confidential comments to the editors, which will not be published.

Choose an item.

To further support our reviewers, we have joined with Publons, where you can gain additional credit to further highlight your hard work (see: https://publons.com/journal/530/gigascience). On publication of this paper, your review will be automatically added to Publons, you can then choose whether or not to claim your Publons credit. I understand this statement.

Yes Choose an item.
