# Supplementary material for: metaXplor: an interactive viral and microbial metagenomic data manager
Source: Gigascience. 2021 Feb 2;10(2):giab001. doi: 10.1093/gigascience/giab001 (PMC7931823; doi:10.1093/gigascience/giab001)
Supplement: giab001_Reviewer_1_Report_Revision_1 — Christopher Hunter, Ph.D -- 11/30/2020 Reviewed [file giab001_reviewer_1_report_revision_1.pdf]

Reviewer Report

Title: metaXplor: an interactive viral and microbial metagenomic data manager

Version: Revision 1 **Date:** 11/30/2020

Reviewer name: Christopher Hunter, Ph.D.

Reviewer Comments to Author:

The authors have addressed all of my recommendations adequately and I believe the changes have made the tool and manuscript an even more useful tool that will be welcomed by the community.
I did encounter an odd message on the app that should be looked at, I believe its just a missing bit of text;
on https://metaxplor.cirad.fr/metaXplor/main.jsp I clicked "Explore data", then "search" without changing any of the values/dropdowns just as it appears by default.
Then I clicked the Taxonomy tree view icon, when I selected "BlastX" from the dropdown menu a dialog box popped onto the screen saying just "metaxplor.cirad.fr says" with nothing else, and no content to the page. I'm guessing that there are no blastx results to be displayed, but the dialog box doesn't say anything?! When I change the dropdown back to BlastN it all works fine again.
That minor bug aside, the tool works as expected, as with all user interfaces there is always room for improvement and personal preferences mean you cannot please everyone all of the time, but in general it is a clean look and the different functionalities can be discovered and understood in the documentation.
I have no problem in recommending this manuscript for publication, and I thank the authors for their consideration and addressing all of my previous review points.

Level of Interest

Please indicate how interesting you found the manuscript: Choose an item.

Quality of Written English

Please indicate the quality of language in the manuscript: Choose an item.

Declaration of Competing Interests

Please complete a declaration of competing interests, considering the following questions:

- Have you in the past five years received reimbursements, fees, funding, or salary from an organisation that may in any way gain or lose financially from the publication of this manuscript, either now or in the future?

- Do you hold any stocks or shares in an organisation that may in any way gain or lose financially from the publication of this manuscript, either now or in the future?
- Do you hold or are you currently applying for any patents relating to the content of the manuscript?
- Have you received reimbursements, fees, funding, or salary from an organization that holds or has applied for patents relating to the content of the manuscript?
- Do you have any other financial competing interests?
- Do you have any non-financial competing interests in relation to this paper?

If you can answer no to all of the above, write 'I declare that I have no competing interests' below. If your reply is yes to any, please give details below.

I am a GigaScience employee. Additionally, I am a volunteer member of the Genomics Standards Consortium.

I agree to the open peer review policy of the journal. I understand that my name will be included on my report to the authors and, if the manuscript is accepted for publication, my named report including any attachments I upload will be posted on the website along with the authors' responses. I agree for my report to be made available under an Open Access Creative Commons CC-BY license (http://creativecommons.org/licenses/by/4.0/). I understand that any comments which I do not wish to be included in my named report can be included as confidential comments to the editors, which will not be published.

Choose an item.

To further support our reviewers, we have joined with Publons, where you can gain additional credit to further highlight your hard work (see: https://publons.com/journal/530/gigascience). On publication of this paper, your review will be automatically added to Publons, you can then choose whether or not to claim your Publons credit. I understand this statement.

Yes Choose an item.
