# Supplementary material for: metaXplor: an interactive viral and microbial metagenomic data manager
Source: Gigascience. 2021 Feb 2;10(2):giab001. doi: 10.1093/gigascience/giab001 (PMC7931823; doi:10.1093/gigascience/giab001)
Supplement: giab001_Reviewer_2_Report_Original_Submission — Gregory Vey, Ph.D. -- 8/8/2020 Reviewed [file giab001_reviewer_2_report_original_submission.pdf]

Reviewer Report

Title: metaXplor: an interactive viral and microbial metagenomic data manager

Version: Original Submission **Date:** 8/8/2020

Reviewer name: Gregory Vey, Ph.D.

Reviewer Comments to Author:

Background
Generally, there is a lack of citation for the opening assertions and a rather arbitrary citation list for landmark works. In particular, the Background does not stipulate the domain-specific problem that this tool proposes to solve or improve. A review of similar and/or competing resources, or the lack thereof, would help to better characterize the contribution of the present work ad help to avoid potential criticism of a derivative connection to Gigwa v2.
Architecture and data model
Application architecture outline
I am impressed by the authors' decision to use an industry standard software development paradigm. I feel that it would further strengthen this work to briefly remark on the durability, maintainability, and extendibility of their implementation, as a consequence of having elected to use freely available and well-accepted standards, such as the Spring Framework and Apache Tomcat. In my opinion, this approach lends significant credibility to the application architecture and is a refreshing change in a research domain presently inundated by ad hoc python scripting.
Data model
I can appreciate the decision to use NoSQL and the Data Model is well illustrated in Figure 5. However, given the prevalence of relational database management systems, it is necessary to briefly explain and justify the choice to use NoSQL in the present work, which is assumed to be motivated by the need for a schemaless model. Also, the author's do describe connections to NCBI taxonomy, BLAST, etc. but do not explicitly stipulate if the data model aligns to a specific metadata standard for its respective domain. A more explicit discussion of how this tool relates to FAIR standards, or even just interoperability in general, would benefit this work.

Level of Interest

Please indicate how interesting you found the manuscript: Choose an item.

Quality of Written English

Please indicate the quality of language in the manuscript: Choose an item.

Declaration of Competing Interests

Please complete a declaration of competing interests, considering the following questions:

- Have you in the past five years received reimbursements, fees, funding, or salary from an organisation that may in any way gain or lose financially from the publication of this manuscript, either now or in the future?
- Do you hold any stocks or shares in an organisation that may in any way gain or lose financially from the publication of this manuscript, either now or in the future?
- Do you hold or are you currently applying for any patents relating to the content of the manuscript?
- Have you received reimbursements, fees, funding, or salary from an organization that holds or has applied for patents relating to the content of the manuscript?
- Do you have any other financial competing interests?
- Do you have any non-financial competing interests in relation to this paper?

If you can answer no to all of the above, write 'I declare that I have no competing interests' below. If your reply is yes to any, please give details below.

I declare that I have no competing interests

I agree to the open peer review policy of the journal. I understand that my name will be included on my report to the authors and, if the manuscript is accepted for publication, my named report including any attachments I upload will be posted on the website along with the authors' responses. I agree for my report to be made available under an Open Access Creative Commons CC-BY license (http://creativecommons.org/licenses/by/4.0/). I understand that any comments which I do not wish to be included in my named report can be included as confidential comments to the editors, which will not be published.

Choose an item.

To further support our reviewers, we have joined with Publons, where you can gain additional credit to further highlight your hard work (see: https://publons.com/journal/530/gigascience). On publication of this paper, your review will be automatically added to Publons, you can then choose whether or not to claim your Publons credit. I understand this statement.

Yes Choose an item.
