# Supplementary material for: metaXplor: an interactive viral and microbial metagenomic data manager
Source: Gigascience. 2021 Feb 2;10(2):giab001. doi: 10.1093/gigascience/giab001 (PMC7931823; doi:10.1093/gigascience/giab001)
Supplement: giab001_Reviewer_3_Report_Original_Submission — Justine Debelius -- 8/11/2020 Reviewed [file giab001_reviewer_3_report_original_submission.pdf]

Reviewer Report

Title: metaXplor: an interactive viral and microbial metagenomic data manager

Version: Original Submission **Date:** 8/11/2020

Reviewer name: Justine Debelius

Reviewer Comments to Author:

SempÃ©rÃ© et al present metaXplor, an interactive database for microbiome and viral data. The authors claim that current available platforms fail to meet the needs of multiple study management, data querying work, and meta analysis. metaXplor is built on a mongoDB backend, which uses an Oracle database to facilitate blast searches and computationally intensive tasks. A docker container is available, but it is unclear whether the docker container requires an internet connection - i.e. does metaXplor require this oracle backend to function. For users, the database accepts sample information, an already computed table, a fasta of feature sequences and taxonomy. The results produced include a krona plot, and a map view of the results.

One of the major issues that remains unaddressed in the manuscript is how this compares to the qiita database and associated infrastructure - a comprehensive database built for storing and analyzing studies.

I would also like to try the interface, which I assume is what most end users will use. Is it possible for the authors to provide an anonymous reviewer login to explore/test the system?

Level of Interest

Please indicate how interesting you found the manuscript: Choose an item.

Quality of Written English

Please indicate the quality of language in the manuscript: Choose an item.

Declaration of Competing Interests

Please complete a declaration of competing interests, considering the following questions:

- Have you in the past five years received reimbursements, fees, funding, or salary from an organisation that may in any way gain or lose financially from the publication of this manuscript, either now or in the future?
- Do you hold any stocks or shares in an organisation that may in any way gain or lose financially from the publication of this manuscript, either now or in the future?
- Do you hold or are you currently applying for any patents relating to the content of the manuscript?

- Have you received reimbursements, fees, funding, or salary from an organization that holds or has applied for patents relating to the content of the manuscript?
- Do you have any other financial competing interests?
- Do you have any non-financial competing interests in relation to this paper?

If you can answer no to all of the above, write 'I declare that I have no competing interests' below. If your reply is yes to any, please give details below.

I declare that I have no competing interests

I agree to the open peer review policy of the journal. I understand that my name will be included on my report to the authors and, if the manuscript is accepted for publication, my named report including any attachments I upload will be posted on the website along with the authors' responses. I agree for my report to be made available under an Open Access Creative Commons CC-BY license (http://creativecommons.org/licenses/by/4.0/). I understand that any comments which I do not wish to be included in my named report can be included as confidential comments to the editors, which will not be published.

Choose an item.

To further support our reviewers, we have joined with Publons, where you can gain additional credit to further highlight your hard work (see: https://publons.com/journal/530/gigascience). On publication of this paper, your review will be automatically added to Publons, you can then choose whether or not to claim your Publons credit. I understand this statement.

Yes Choose an item.
